# Characterization of 2-phenanthroyl-CoA reductase, an ATP-independent type III aryl-CoA reductase involved in anaerobic phenanthrene degradation

**DOI:** 10.1128/aem.00166-25

**Published:** 2025-04-17

**Authors:** Nadia A. Samak, Frederik Götz, Khadija Adjir, Torsten Schaller, Marvin Häßler, Oliver J. Schmitz, Jonas Fax, Gebhard Haberhauer, Alina Surmeneva, Rainer U. Meckenstock

**Affiliations:** 1 Environmental Microbiology and Biotechnology (EMB), Faculty of Chemistry, University of Duisburg-Essen 27170, Essen, Germany; 2 Laboratory of Thermodynamics and Molecular Modeling, Faculty of Chemistry, USTHB 61758 , Algiers, Algeria; 3 Organic Chemistry, Faculty of Chemistry, University of Duisburg-Essen 27170, Essen, Germany; 4 Applied Analytical Chemistry, Faculty of Chemistry, University of Duisburg-Essen 27170, Essen, Germany; Shanghai Jiao Tong University, Shanghai, China

**Keywords:** reductase, old-yellow enzyme, 2-phenanthroyl-CoA, anaerobic phenanthrene degradation, PAHs, culture TRIP_1

## Abstract

Anaerobic degradation of polycyclic aromatic hydrocarbons (PAHs) with three or more aromatic rings is extremely slow because the compounds are very poorly soluble in water and chemically stable. Phenanthrene is the only three-ring PAH where the anaerobic degradation has been partially elucidated. Phenanthrene is first activated via carboxylation producing 2-phenanthroate, which is further converted to 2-phenanthroyl-coenzyme A (CoA) via the enzyme 2-phenanthroate:CoA ligase. In this study, we elucidated the next degradation step, the reduction of 2-phenanthroyl-CoA to dihydro-2-phenanthroyl-CoA. We cloned the putative gene from the genome of culture TRIP_1 and heterologously expressed and purified the 2-phenanthroyl-CoA reductase enzyme from *Escherichia coli*. The identified monomeric flavo-enzyme belongs to the novel group of type III aryl-CoA reductases in the old-yellow enzyme family and has a molecular mass of 72 kDa. 2-Phenanthroyl-CoA reductase contains one FMN, one FAD, and one [4Fe-4S] iron-sulfur cluster as cofactors. The enzyme has a specific activity of 17.6 ± 0.4 nmol/min/mg, a *K*_m_ value of 1.8 µM, and a Vmax of 7.9 µmol/min/mg at pH 7.5, when reduced methyl viologen was used as electron donor. 2-Phenanthroyl-CoA reductase catalyzed a two-electron reduction step producing one of five possible isomers. Quantum mechanical calculations and nuclear magnetic resonance analysis of the reaction product suggested 9,10-dihydro-2-phenanthroyl-CoA as the most stable isomer. However, our experimental evidence suggests 7,8-dihydro-2-phenanthroyl-CoA (International Union of Pure and Applied Chemistry [IUPAC]: 1,2-dihydro-7-phenanthroyl-CoA) or 5,6-dihydro-2-phenanthroyl-CoA (IUPAC: 3,4-dihydro-7-phenanthroyl-CoA) as the most likely reduced product with a saturated bond in ring 3 of the substrate 2-phenanthroyl-CoA, before undergoing isomerization changes to reach the more stable structure of 9,10-dihydro-2-phenanthroyl-CoA.

**IMPORTANCE:**

PAHs are a group of highly toxic and persistent environmental pollutants. The anaerobic degradation of three-ring PAHs like phenanthrene is still poorly understood. Phenanthrene degradation starts with a carboxylation reaction to form 2-phenanthroic acid followed by a CoA-thioesterification reaction catalyzed by 2-phenanthroate:CoA ligase to produce 2-phenanthroyl-CoA. The next degradation step is the reduction of 2-phenanthroyl-CoA to dihydro-2-phenanthroyl-CoA to overcome the resonance energy of the aromatic ring system. Herein, we elucidated that the reduction reaction is catalyzed by the enzyme 2-phenanthroyl-CoA reductase. Furthermore, we provided biochemical and structural properties of the heterologously expressed and purified 2-phenanthroyl-CoA reductase, which confirmed that the enzyme belongs to the novel group of type III aryl-CoA reductases in the old-yellow enzyme family.

## INTRODUCTION

Polycyclic aromatic hydrocarbons (PAHs) are threatening humans and the environment since they are highly toxic and carcinogenic (1). PAHs spread into the environment through oil and gasoline spills, incomplete combustion of coal and wood, or exhaust from, e.g., domestic heaters or industry. The hydrophobic compounds adsorb to particulate organic and inorganic matter, leading to deposition in sediments where higher organic loads cause rapid oxygen consumption, turning the condition into anoxic (2, 3). Hence, PAHs especially accumulate in anoxic sediments, where they stay recalcitrant due to the poor solubility in water and the connected limited bioavailability, as well as the lack of molecular oxygen as electron acceptor and cosubstrate (4).

Aerobic microorganisms can utilize molecular oxygen as a cosubstrate for mono- or dioxygenases. These enzymes usually contain metal or flavin/pterin cofactors that are directly involved in the reductive activation of dioxygen for an oxidative attack of the aromatic ring (5, 6). Anaerobic microorganisms, however, cannot make use of the reactive molecular oxygen and have developed different strategies for the activation of PAHs and for overcoming the resonance energy of the aromatic rings (3, 7, 8).

Anaerobic degradation of polycyclic aromatic hydrocarbons has to cope with three biochemical problems: the activation of the chemically inert compound, overcoming the resonance energy of the aromatic ring system, and cleavage of the rings to channel smaller fatty acids into the central metabolism. Naphthalene is the smallest PAH with two aromatic rings and is considered a model compound for PAH degradation (9). Two different ways of the anaerobic activation of naphthalene are known so far. Methylnaphthalene is activated by fumarate addition, similar to anaerobic toluene degradation (10, 11). The non-substituted PAH naphthalene is activated through carboxylation to 2-naphthoate by naphthalene carboxylase (12–14) followed by coenzyme A (CoA) ester formation through 2-naphthoate:CoA ligase (3, 15–17). Then, 2-naphthoyl-CoA is transformed via reductive de-aromatization with 2-naphthoyl-CoA reductase and dihydro-2-naphthoyl-CoA reductase producing 5,6,7,8-tetrahydro-2-naphthoyl-CoA (18–20). These two enzymes constitute the first examples of the new class of type III aryl-CoA reductases that are ATP-independent flavo-enzymes belonging to the old-yellow enzyme family. Reduction of the remaining aromatic ring I of tetrahydro-2-naphthoyl-CoA is performed by an ATP-dependent type I aryl-CoA reductase producing hexahydro-2-naphthoyl-CoA, similar to benzoyl-CoA reductase (17, 21, 22). Ring cleavage is then initiated by β-oxidation-like reactions. The so far available data suggest that anaerobic degradation of bigger PAHs with three rings follows a similar strategy of carboxylation, ring reduction, and ring cleavage with beta oxidation-like reactions (3, 13, 23–25).

We enriched the sulfate-reducing culture TRIP_1, which grows with the three-ring PAH phenanthrene as the sole carbon and electron source and sulfate as electron acceptor, leading to a complete degradation of phenanthrene to CO_2_ (24). Similar to naphthalene, phenanthrene is activated by carboxylation to 2-phenanthroic acid (24, 25), which is further converted to 2-phenanthroyl-CoA by the ATP-dependent enzyme 2-phenanthroate:CoA ligase (26). In analogy to anaerobic naphthalene degradation, the phenanthrene degradation pathway was proposed to continue with several consecutive two-electron reduction steps to break the aromaticity of the ring system, catalyzed by four putative type III aryl-CoA reductases encoded in the genome of TRIP_1 (24, 25). The putative reductases share approximately 35% homology with 2-naphthoyl-CoA reductase from the Deltaproteobacteria N47 and NaphS2 (25). The transcriptome/proteome analysis of culture TRIP_1 revealed that the genes encoding the oxidoreductases are surrounded by genes encoding putative hydratases, dehydrogenases, hydrolases, and thiolases, which could be responsible for phenanthrene ring opening by β-oxidation after the aromatic ring reduction, similar to anaerobic degradation of naphthalene (25).

In this study, we aimed at further elucidating the anaerobic degradation pathway of bigger PAHs with three or more aromatic rings. We characterized 2-phenanthroyl-CoA reductase, which catalyzes the first reduction step of the phenanthrene aromatic ring system. The gene was heterologously expressed in *Escherichia coli* Bl21(DE3); the enzyme was purified and characterized; and the reaction was elucidated.

## RESULTS

### Genomic localization of 2-phenanthroyl-CoA reductase and homology with other reductases

**Fig 1:**
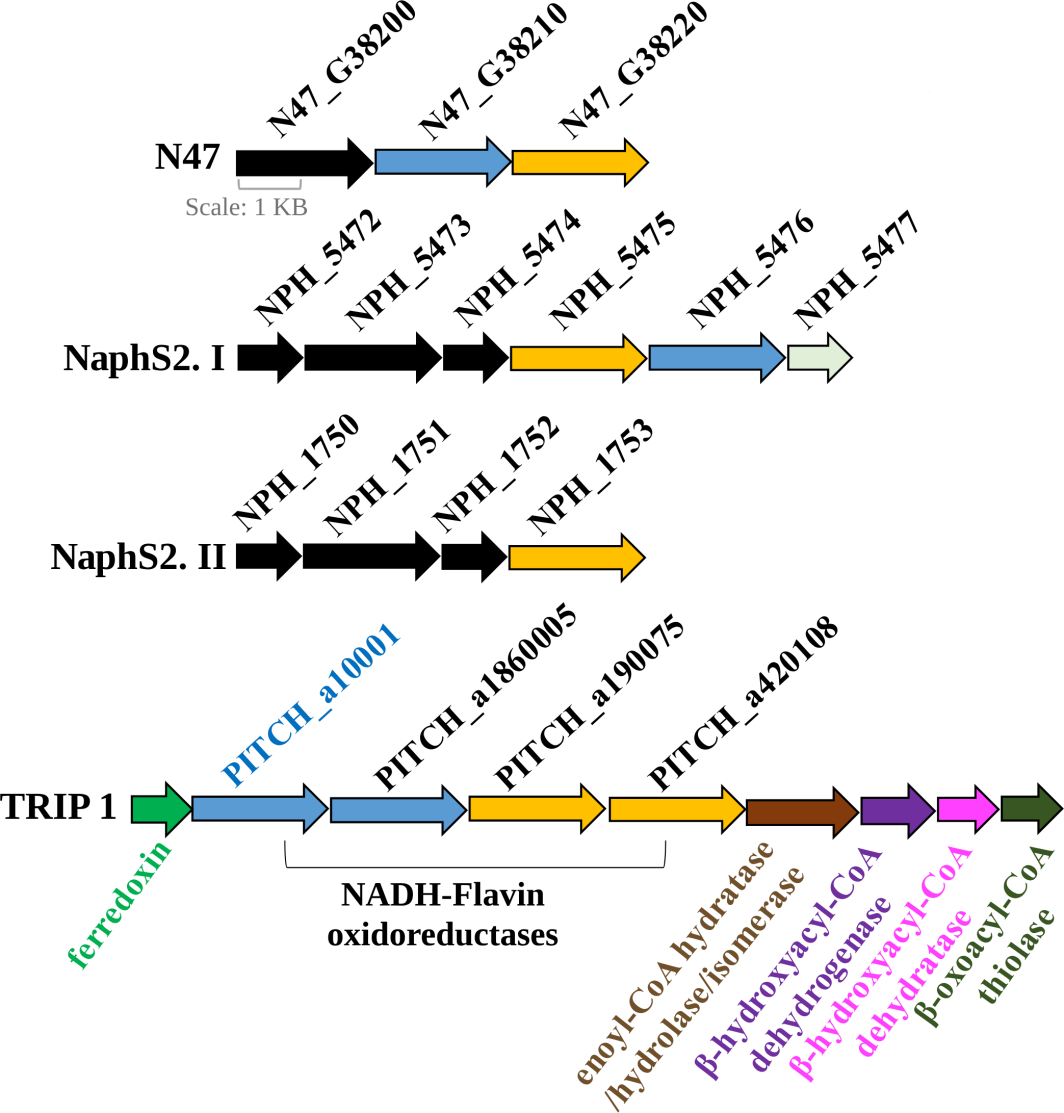
Comparison of the location of the 2-phenanthroyl-CoA reductase gene (PITCH_a10001) in the metagenome of culture TRIP_1 and the homologous genes in the metagenomes of the naphthalene-degrading cultures N47 and NaphS2. The genes in cultures N47 (N47_G38210, 5,6-dihydro-2-naphthoyl-CoA reductase) and NaphS2 (NPH_5476, 5,6-dihydro-2-naphthoyl-CoA reductase) that are similar to the 2-phenanthroyl-CoA reductase gene (PITCH_a10001 and PITCH_a1860005) in TRIP_1 are presented as blue arrows. Yellow arrows represent the homology between the 2-naphthoyl-CoA reductase gene in cultures N47 (N47_G38220), NaphS2 (NPH_5475 and NPH_1753), and culture TRIP_1 (PITCH_a190075 and PITCH_a420108). The image also indicates the localization of the oxidoreductase genes in the context of putative genes for β-oxidation (enoyl-CoA hydratase/hydrolase/isomerase, β-hydroxyacyl-CoA dehydrogenase, β-hydroxyacyl-CoA dehydratase, and β-oxoacyl-CoA thiolase).

In this study, we focused on the characterization of 2-phenanthroyl-CoA reductase, which is responsible for the first reduction step of 2-phenanthroyl-CoA producing dihydro-2-phenanthroyl-CoA. The PITCH_a10001 gene encoding 2-phenanthroyl-CoA reductase was one out of four genes potentially encoding NADH-flavin oxidoreductases in the metagenome of the sulfate-reducing enrichment culture TRIP_1. The amino acid sequence of 2-phenanthroyl-CoA reductase showed high similarity with oxidoreductases of the old-yellow enzyme family from cultures N47 and NaphS2. 2-Phenanthroyl-CoA reductase showed 31%–32% amino acid similarity with 2-naphthoyl-CoA reductase encoded by the genes N47_G38220, NPH_5475, and NPH_5473 (20) and 32%–34% amino acid similarity with 5,6-dihydro-2-naphthoyl-CoA reductase encoded by the genes N47_G38210 and NPH_5476 (Fig. 1) (27, 28).

### Heterologous expression and purification of 2-phenanthroyl-CoA reductase

**Fig 2:**
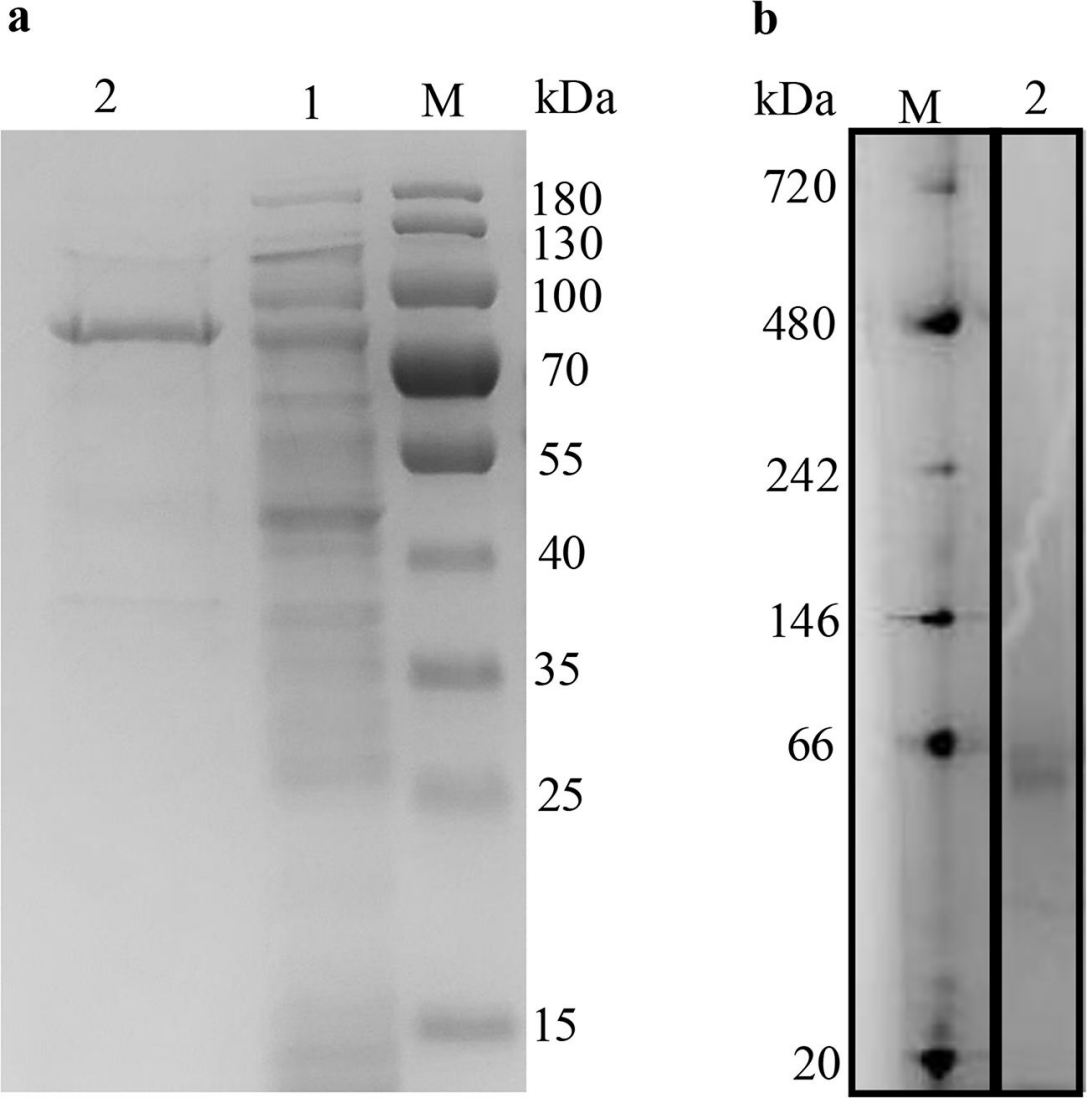
SDS-PAGE (**a**) and NATIVE-PAGE analysis (**b**) of 2-phenanthroyl-CoA reductase produced from the recombinant plasmid PASG-IBA103-PITCH_a10001. (**a**) M, protein MW standard; lane 1, *E. coli* cell-free extract; lane 2, 2-phenanthroyl-CoA reductase after purification with a Strep-Tactin resin. (**b**) M, protein MW standard; lane 2, purified 2-phenanthroyl-CoA reductase in blue native gel.

The PITCH_a10001 gene, putatively encoding 2-phenanthroyl-CoA reductase, was heterologously expressed in *E. coli* and tested for 2-phenanthroyl-CoA reduction. Soluble 2-phenanthroyl-CoA reductase was expressed from *E. coli* carrying the recombinant plasmid PASG-IBA103-PITCH_a10001 with a C-terminal twin-Strep-tag under oxic conditions. From 5 g wet mass of *E. coli* cells, 1.3–1.5 mg protein was produced. Sodium dodecylsulfate-polyacrylamide gel electrophoresis (SDS-PAGE) analysis confirmed the purity of the expressed enzyme and showed a molecular mass of about 72 kDa (Fig. 2a). Blue native gels also showed a mass of ≈72 kDa, indicating a monomeric structure of the produced 2-phenanthroyl-CoA reductase (Fig. 2b).

### Characterization of 2-phenanthroyl-CoA reductase

**Fig 3:**
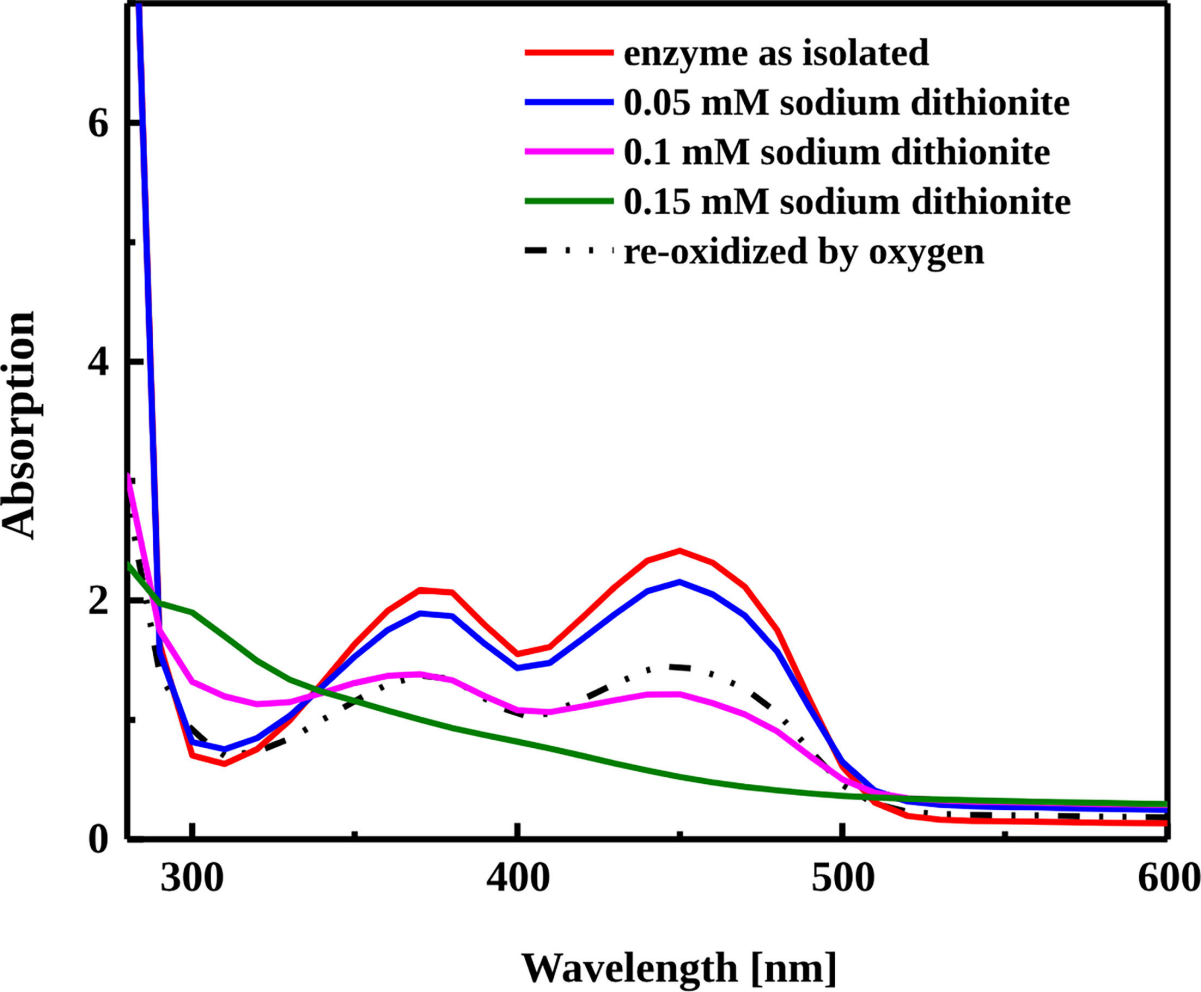
UV/vis spectra of the purified 2-phenanthroyl-CoA reductase as isolated and after reduction of the enzyme in steps of 0.05 mM sodium dithionite each. Complete reduction was achieved with 0.15 mM sodium dithionite, and the enzyme was re-oxidized by exposure to oxygen.

The gene sequence indicated that 2-phenanthroyl-CoA reductase belongs to the old-yellow enzyme family, which was supported by the slightly yellowish color, indicating the presence of flavin cofactors. The UV/visible (UV/vis) spectrum confirmed the presence of flavin cofactors with two characteristic peaks at 375 and 450 nm for neutral FMN and FAD (Fig. 3). The stepwise addition of 0.05 mM sodium dithionite showed a gradual depletion of the peaks until complete reduction was achieved by 0.15 mM sodium dithionite, indicating that 2-phenanthroyl-CoA reductase was isolated in the oxidized form. The reoxidation of the reduced enzymes was simply performed by exposing the solution to oxygen, reestablishing the characteristic peaks of the flavin in the UV-vis spectra (Fig. 3). The FMN and FAD content were measured by extraction and quantification with liquid chromatography/mass spectrometry (LC/MS) and compared with commercial standards, giving an average flavin content of 0.4 ± 0.2 FMN/monomer and 1.1 ± 0.3 FAD/monomer, respectively (Table 1), indicating one FMN and one FAD per monomer. To examine if the enzyme contains an iron-sulfur cluster, the iron content was measured colorimetrically, resulting in an average value of 3.6 ± 0.3 Fe/monomer, indicating one 4Fe-4S cluster per monomer.

**TABLE 1:** Biochemical characteristics of 2-phenanthroyl-CoA reductase

Characteristic	Value
Specific activity (nmol/min/mg)	17.6 ± 0.4
*K*_M_ (μM)	1.8
Vmax (μmol/min/mg)	7.9
FMN content	0.4 ± 0.2 FMN/monomer
FAD content	1.1 ± 0.3 FAD/monomer
Iron content	3.6 ± 0.3 Fe/monomer
Used electron donors (activity given in percentage of the maximal activity measured with reduced methyl viologen)	Dithionite-reduced methyl viologen: 100% Dithionite: 20% NADH: 25% Ti(III)-citrate: 0% NAD(P)H: 0%
Oxygen sensitive	Yes

### Structure modeling of 2-phenanthroyl-CoA reductase

To further analyze the structure and mechanism of the 2-phenanthroyl-CoA reductase (PITCH_a10001), 2-naphthoyl-CoA reductase (N47_G38220) from the enrichment culture N47 was used as a model enzyme. The sequence alignment of the two genes showed 31% identity and 52% similarity (Fig. S2). The four cysteines that coordinate the iron-sulphur cluster in 2-naphthoyl-CoA reductase were conserved in the amino acid sequence of 2-phenanthroyl-CoA reductase supporting the presence of one 4Fe-4S cluster per 2-phenanthroyl-CoA reductase monomer. Alpha Fold was used to predict the structure of 2-phenanthroyl-CoA reductase because we did not have a crystal structure, so far. Alpha Fill was used to populate the Alpha Fold Protein Structure Data Base protein structure of PITCH_a10001 with the cofactors FAD, FMN, and a 4Fe-4S cluster (29–32). The cofactors had very low interface root mean square deviation (RMSD) scores, and after optimizing with the Yasara software, almost no transplant clash score was observed on AlphaFill (29, 33), indicating that the placement of the transplanted cofactors is realistic. After alignment with pyMol, the two protein structures showed an RMSD of 1.814 Å, indicating very similar secondary and tertiary structures (Fig. S3). Moreover, the cofactors of the two protein structures showed very similar positioning (Fig. S3).

Molecular mechanic (MM) simulation of the modeled complex was performed to analyze the possible cofactor interactions and mechanism of 2-phenanthroyl-CoA reduction. After energy minimization, the protein ligand interactions of the cofactors of the 2-phenanthroyl-CoA reductase protein complex were compared with the cofactors of 2-naphthoyl-CoA reductase.

**Fig 4:**
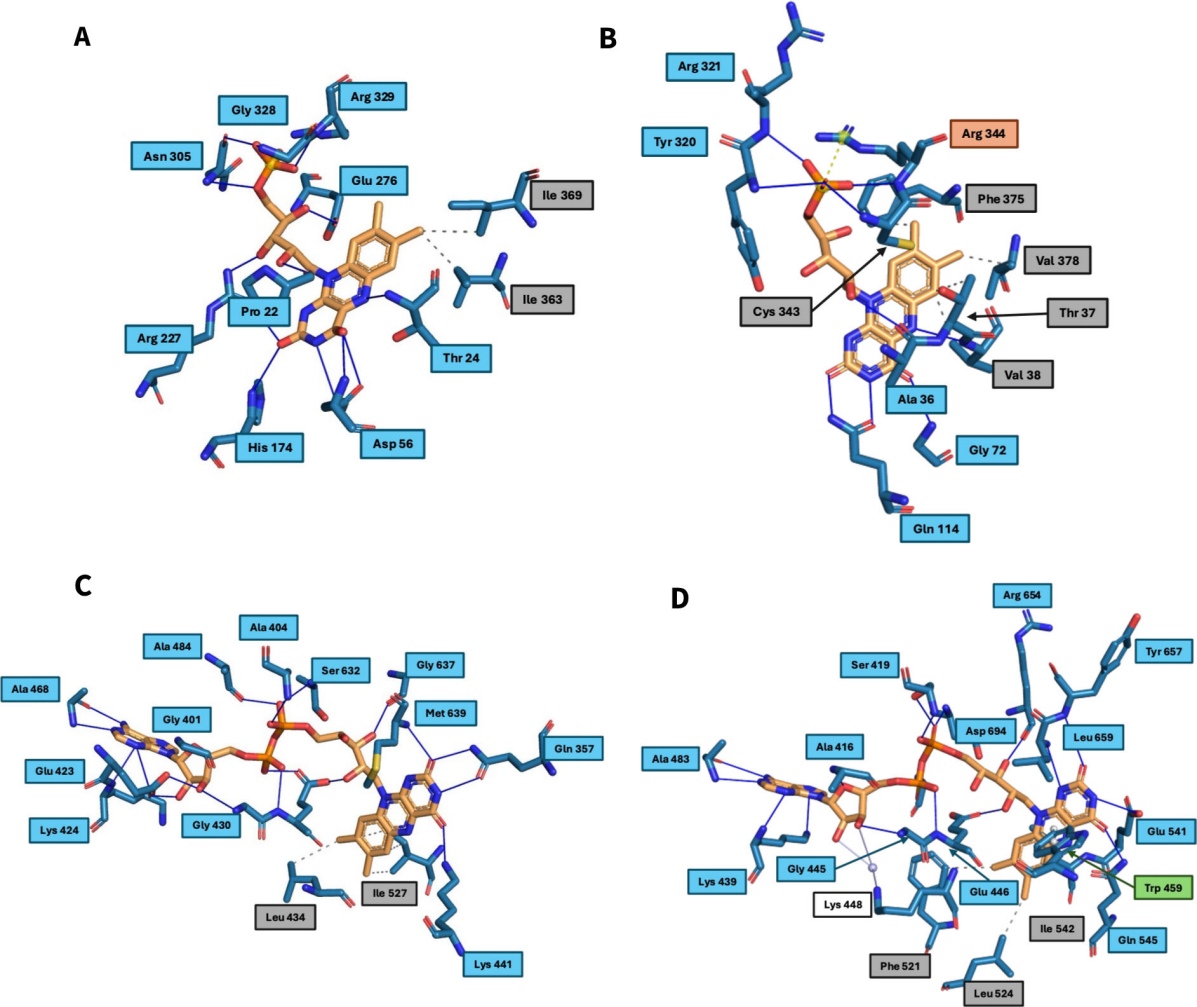
Protein-ligand interaction profile (PLIP) of FMN in (A) 2-phenanthroyl-CoA reductase and (B) 2-naphthoyl-CoA reductase. Blue solid lines show hydrogen bonds; gray dotted lines denote hydrophobic interactions; and yellow/orange dotted lines show salt bridges. PLIP of FAD in (C) 2-phenanthroyl-CoA reductase and (D) 2-naphthoyl-CoA reductase. Blue solid lines show hydrogen bonds; gray dotted lines show hydrophobic interactions; green dotted lines show π-stacking interactions; and white solid lines show water bridges. The labels of the interacting amino acid side chains are colored in the color corresponding to the interaction type (34, 35).

The protein ligand interaction profiling (PLIP) of FMN in the 2-phenanthroyl-CoA reductase modeled structure (Fig. 4A) showed that FMN is similarly coordinated as FMN in 2-naphthoyl-CoA reductase (Fig. 4B), even though there are more hydrogen bonds and less hydrophobic interactions. FAD shows similar coordination patterns, with more prominent hydrogen bond interactions (Fig. 4C). The only big difference between the FAD coordination patterns of the modeled complex (Fig. 4C) and the crystal structure of 2-naphthoyl-CoA reductase is that our simulation did not show any water bridges or π-stacking interactions. The role of the water bridges in 2-naphthoyl-CoA reductase (Fig. 4D) occurs via Glu 423. This means that the transplantation of the FAD and following energy minimization yielded results that are very realistic. Lastly, the PLIP of 2-phenanthroyl-CoA (Fig. S4A) showed that the coordination of the molecule was worse than the coordination of 2-naphthoyl-CoA in 2-naphthoyl-CoA reductase (Fig. S4B). Even though the aromatic ring system got coordinated through hydrophobic interactions with His177, Tyr179, Thr 24, and Ile 363, the CoA part of the molecule had fewer hydrophobic and hydrogen bond interactions. This could mean that either the ligand-receptor interaction in this enzyme is far weaker than that in the 2-naphthoate-CoA reductase or that the modeled binding mode does not correspond to the real binding mode.

### Determination of 2-phenanthroyl-CoA reductase activity

**Fig 5:**
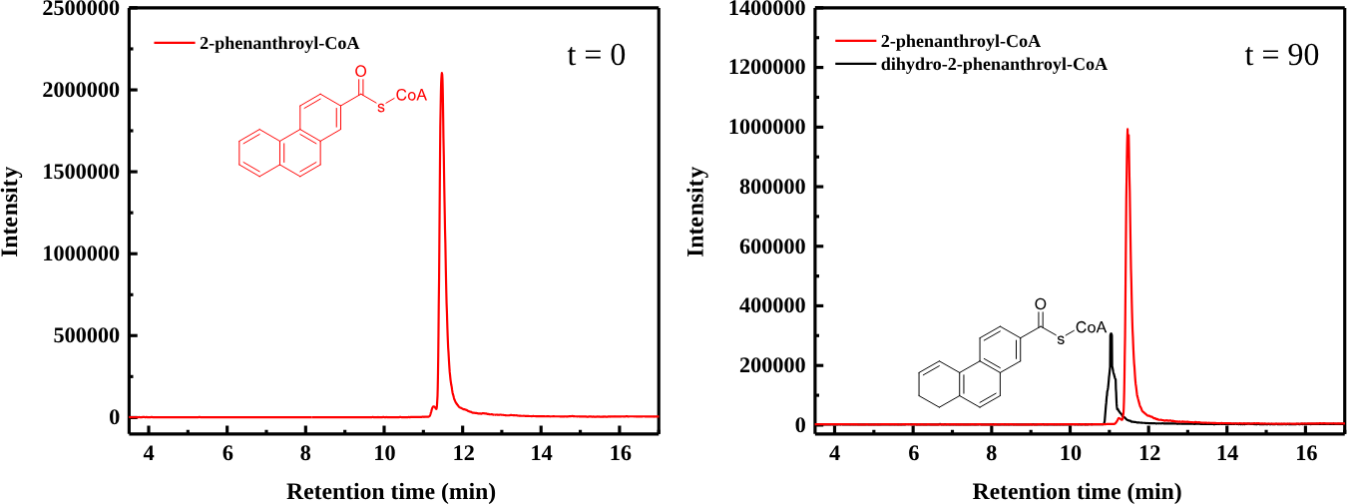
LC/MS chromatograms (Shimadzu single quadrupole mass-spectrometer) showing the time-dependent conversion of 2-phenanthroyl-CoA to the reduced product dihydro-2-phenanthroyl-CoA by purified 2-phenanthroyl-CoA reductase. The red line indicates the substrate 2-phenanthroyl-CoA (*m*/*z* = 972, positive ion mode) and the black line indicates the dihydro-2-phenanthroyl-CoA produced after 90 min (*m*/*z* = 974, positive ion mode). The position of the saturated bond in ring 3 of dihydro-2-phenanthroyl-CoA is not known and only shown exemplarily.

Different electron donors were tested for the best conversion of 2-phenanthroyl-CoA to dihydro-2-phenanthroyl-CoA. 2-Phenanthroyl-CoA reductase catalyzed the conversion of 2-phenanthroyl-CoA (mass-to-charge ratio [*m*/*z*] = 972) to dihydro-2-phenanthroyl-CoA (*m*/*z* = 974) with a specific activity of 17.6 ± 0.4 nmol/min/mg, as detected with LC/MS in positive ion mode (Fig. 5). The initial rate of 2-phenanthroyl-CoA reduction followed Michaelis-Menten kinetics with a Michaelis constant (*K*_*m*_) value of 1.8 µM and a maximal rate of reaction (*V*_max_) of 7.9 µmol/min/mg (Table 1). The reduction reaction required the presence of 1 mM NADH, 50 µM FMN, and 1 mM FAD as essential cofactors and dithionite-reduced methyl viologen (100% activity) as electron donor. A slight reduction activity was also observed when sodium dithionite (20% activity) or NADH (25% activity) was used as the sole electron donor, but no reduction activity was detected with Ti(III)-citrate or NADPH as electron donor. The reduction reaction was ATP-independent, supporting that 2-phenanthroyl-CoA reductase belongs to the ATP-independent class III of aryl-CoA reductases.

The reduction assay was also performed under oxic conditions to determine if the reduction enzymatic reaction is oxygen sensitive. Unlike the reduction of 2-naphthoyl-CoA by 2-naphthoyl-CoA reductase, the reduction of 2-phenanthroyl-CoA by 2-phenanthroyl-CoA reductase was sensitive to oxygen. However, this could also be due to the oxygen sensitivity of the applied electron carrier methyl viologen. 2-Phenanthroyl-CoA reductase was expressed under oxic conditions; therefore, the enzyme must be flushed with nitrogen gas before performing the reduction enzymatic assay.

### Identification of the reduction product, dihydro-2-phenanthroyl-CoA

**Fig 6:**
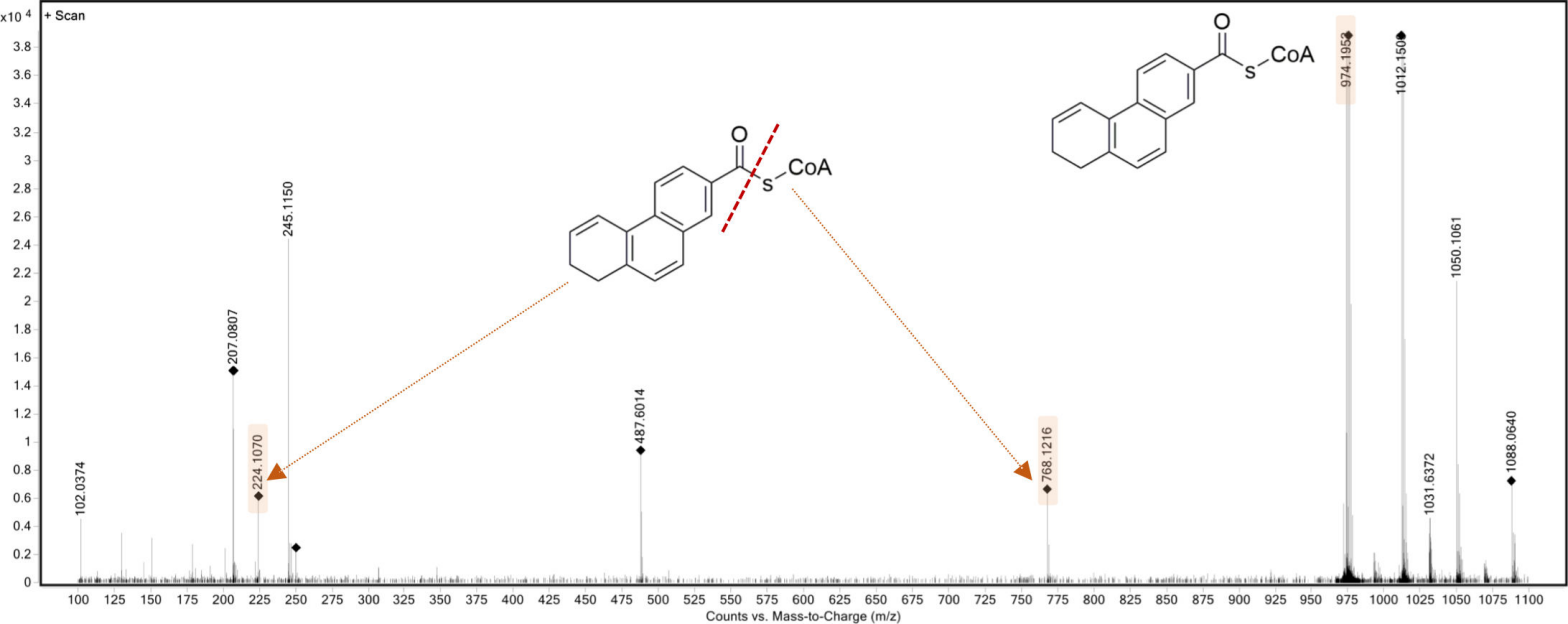
High-resolution mass spectrometry spectrum of the product dihydro-2-phenanthroyl-CoA (*m*/*z* = 974) in positive ion mode showing the fragmentation of the compound to coenzyme A (*m*/*z* = 768, the right side of the cleavage mark) and dihydro-2-phenanthroic acid (*m*/*z* = 224, the left side of the cleavage mark). The position of the saturated bond in ring 3 of dihydro-2-phenanthroyl-CoA is not known and only shown exemplarily.

Liquid chromatography–quadrupole time-of-flight mass spectrometry confirmed the reduction product as C_15_H_11_O-CoA, dihydro-2-phenanthroyl-CoA with an *m*/*z* value of 974 in positive ion mode. The fragmentation of dihydro-2-phenanthroyl-CoA showed two peaks for the free dihydro-2-phenanthroic acid (*m*/*z* = 224) and coenzyme A (*m*/*z* = 768) (Fig. 6).

**Fig 7:**
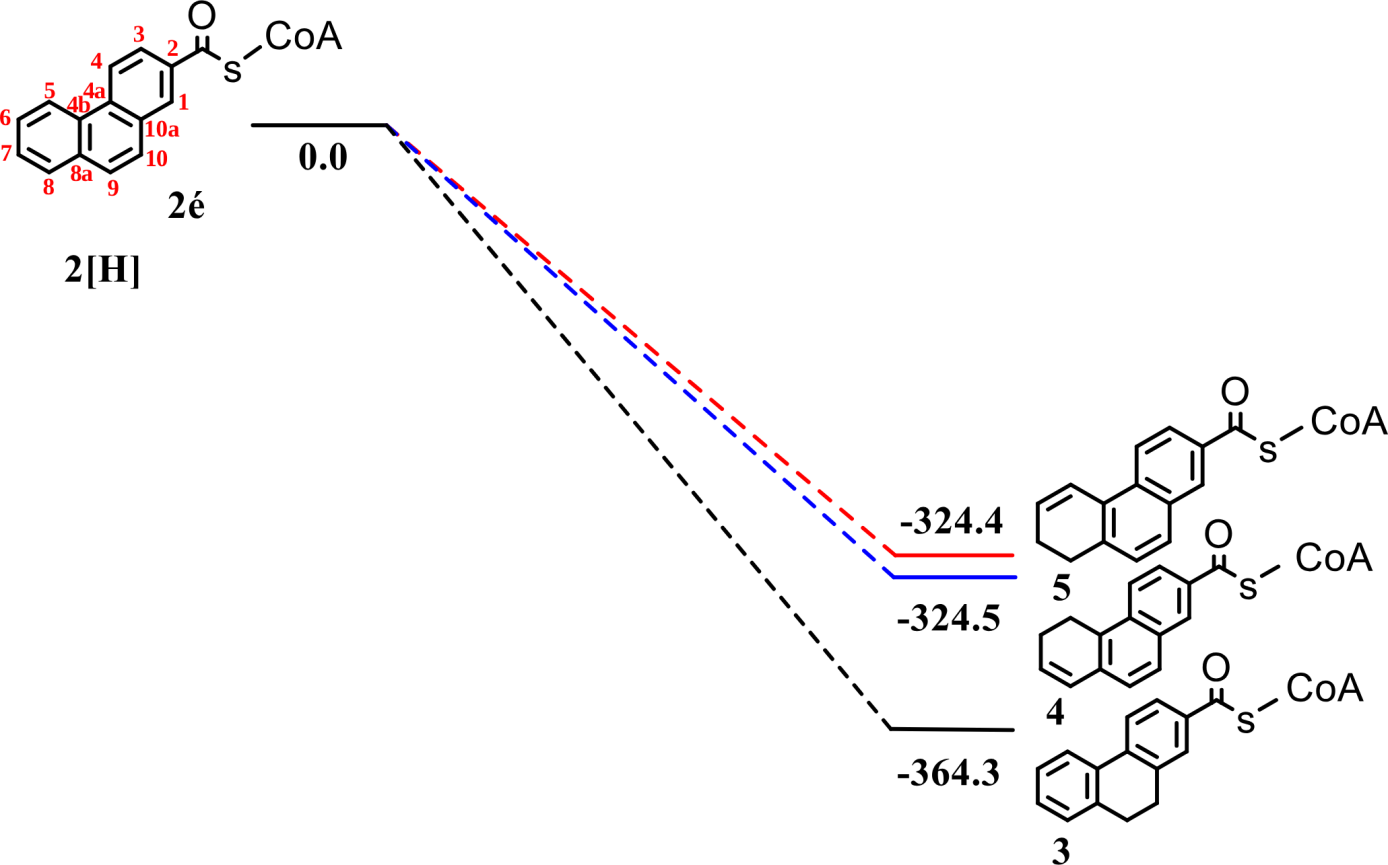
Calculated energies (in kilojoules per mole) of the three possible dihydro-2-phenanthroyl-CoA isomers relative to the starting materials were calculated using B3LYP(CPCM)/6–311 + G(d,p). Isomer **3**, 9,10-dihydro-2-phenanthroyl-CoA; isomer **4**, 5,6-dihydro-2-phenanthroyl-CoA (IUPAC: 3,4-dihydro-7-phenanthroyl-CoA); and isomer **5**, 7,8-dihydro-2-phenanthroyl-CoA (IUPAC: 1,2-dihydro-7-phenanthroyl-CoA).

Theoretical calculations were carried out to predict the most energy-stable isomer of the reduced product, dihydro-2-phenanthroyl-CoA. The relative energies (Δ*E*) of isomers **3**, **4**, and **5** were calculated and indicated that isomer **3** (9,10-dihydro-2-phenanthroyl-CoA) has the most stable structure compared to isomers **4** (5,6-dihydro-2-phenanthroyl-CoA) (International Union of Pure and Applied Chemistry [IUPAC]: 3,4-dihydro-7-phenanthroyl-CoA) and **5** (7,8-dihydro-2-phenanthroyl-CoA) (IUPAC: 1,2-dihydro-7-phenanthroyl-CoA) (Fig. 7). However, these findings do not contradict the hypothesis that isomers **4** and **5** may undergo isomerization changes to reach the more stable structure, isomer **3** (9,10-dihydro-2-phenanthroyl-CoA).

In order to experimentally support the results from the theoretical calculations, nuclear magnetic resonance (NMR) experiments were performed to identify the enzymatically produced and purified isomer of dihydro-2-phenanthroyl-CoA. The sample was hydrolyzed from the CoA-ester form to the free acid, dihydrophenanthrene-2-carboxylic acid, which was then purified. This step had two advantages: first, the NMR spectra were reduced to the significant resonances of the dihydro-2-phenanthroate moiety, and, second, the spectra could be compared with those of a chemically synthesized model substance, the free acid of isomer **3**, 9,10-dihydrophenanthrene-2-carboxylic acid (Fig. 7).

**Fig 8:**
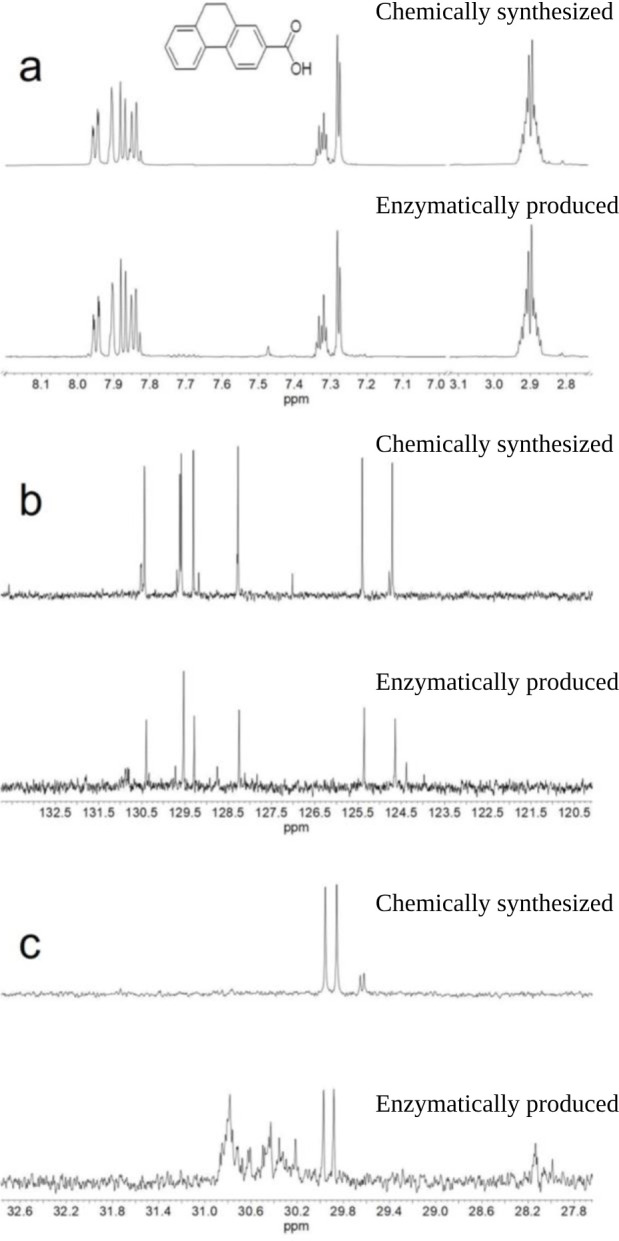
NMR spectra of the carboxylic acid of the isomer obtained by enzymatic reactions (bottom) and chemically synthesized 9,10-dihydrophenanthrene-2-carboxylic acid (top) highlighting their structural identity. (a) ^1^H NMR spectra of the protons on the aromatic rings (7.7–8.0 ppm) and of the methylene protons (2.9 ppm). (b) ^13^C NMR spectra of the protonated carbons in the aromatic rings. Due to the low sample quantity, the signals of the quaternary carbons could not be observed for the enzymatically produced sample. (c) ^13^C NMR spectra of the two methylene carbons (29.9 and 30.0 ppm). Full spectra are shown in the supporting information including a complete assignment of all resonances.

The spectral features (chemical shift and multiplet patterns) of the purified free acid of the product of the reduction reaction of 2-phenanthroyl-CoA agree well with those of the synthesized 9,10-dihydrophenanthrene-2-carboxylic acid (Fig. 8a; see detailed full spectra in Fig. S5 and S6; Table S1). This similarity is also found in the ^13^C NMR spectra in Fig. 8b and c; due to the low sample mass, only the signals of the protonated carbons could be observed. Nevertheless, the combination of ^1^H-^1^H (correlation spectroscopy [COSY] and nuclear Overhauser effect spectroscopy [NOESY]) and ^1^H-^13^C (heteronuclear single quantum coherence spectroscopy [HSQC] and heteronuclear multiple bond correlation spectroscopy [HMBC]) experiments allowed the detection and the full assignment of all resonances. The two-dimensional spectra of the enzymatically produced and the chemically synthesized samples (Fig. S7 and S8) agree as well, confirming that the saturated C-C bond is located in the middle ring of phenanthrene, i.e., isomer **3** (9,10-dihydro-2-phenanthroyl-CoA) has been experimentally found by NMR.

Interestingly, the compound suggested by NMR and theoretical calculations differed from the one analyzed by the UV/vis spectra (Fig. S9). The UV/vis spectra of coenzyme A, the substrate 2-phenanthroyl-CoA, and the product dihydro-2-phenanthroyl-CoA were recorded to confirm the identity of the reduction product (Fig. S9). The product and the substrate spectra showed a small shift compared to coenzyme A, which showed a distinct peak at 250 nm. The substrate phenanthroyl-CoA showed a further broad peak at 260–350 nm, while the product showed a characteristic peak at 250–300 nm, which agreed with the spectra of standards for coenzyme A and the substrate 2-phenanthroyl-CoA. No standard was available for the enzymatically reduced product, dihydro-2-phenanthroyl-CoA, but the spectrum showed a peak at a different position and shape than the signals of coenzyme A and 2-phenanthroyl-CoA. Importantly, the chemically synthesized 9,10-dihydro-2-phenanthroyl-CoA (isomer **3**, Fig. 7) showed a peak shift in the UV/vis spectrum (275–350 nm) compared to the enzymatically reduced product, indicating that the two dihydro-2-phenanthroyl-CoA compounds are two different isomers. This indicates that isomers **4** (5,6-dihydro-2-phenanthroyl-CoA) (IUPAC: 3,4-dihydro-7-phenanthroyl-CoA) and **5** (7,8-dihydro-2-phenanthroyl-CoA) (IUPAC: 1,2-dihydro-7-phenanthroyl-CoA) may undergo isomerization changes to reach the more stable structure, isomer **3** (9,10-dihydro-2-phenanthroyl-CoA).

## DISCUSSION

**Fig 9:**
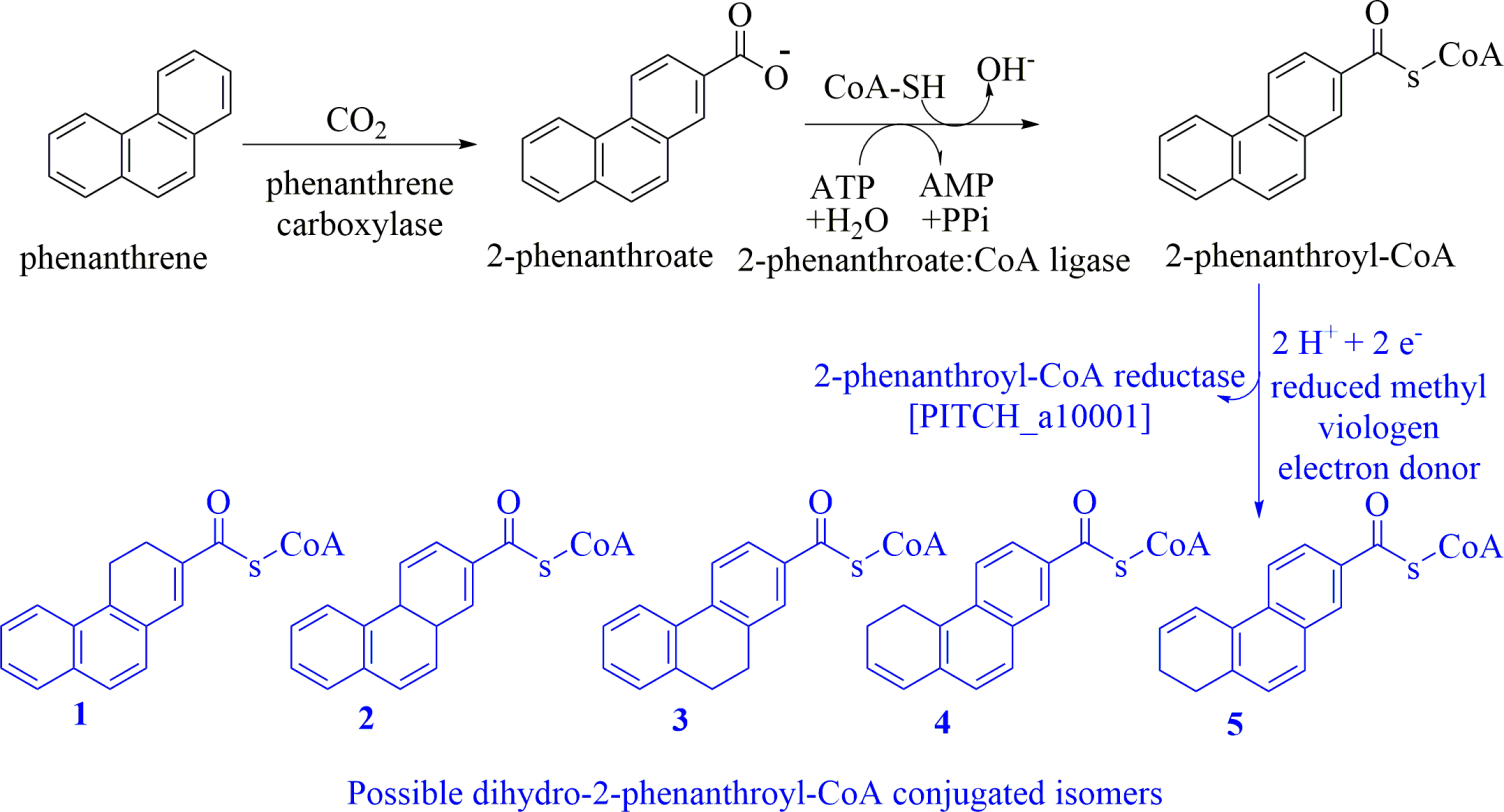
Proposed upper part of the anaerobic phenanthrene degradation pathway showing the elucidated anaerobic degradation reactions of phenanthrene to dihydro-2-phenanthroyl-CoA. Five possible isomers are shown as possible reaction products of 2-phenanthroyl-CoA reductase. Isomer **1**, 3,4-dihydro-2-phenanthroyl-CoA; isomer **2**, 4a,10a-dihydro-2-phenanthroyl-CoA; isomer **3**, 9,10-dihydro-2-phenanthroyl-CoA; isomer **4**, 5,6-dihydro-2-phenanthroyl-CoA (IUPAC: 3,4-dihydro-7-phenanthroyl-CoA); or isomer **5**, 7,8-dihydro-2-phenanthroyl-CoA (IUPAC: 1,2-dihydro-7-phenanthroyl-CoA), are the most likely reduction products. Known enzyme reactions are depicted in black. The enzyme reaction studied here is shown in blue.

In this work, we isolated and characterized the enzyme 2-phenanthroyl-CoA reductase, which is responsible for the reduction of 2-phenanthroyl-CoA to dihydro-2-phenanthroyl-CoA in the anaerobic phenanthrene degradation pathway. Heterologous expression of gene PITCH_a10001 from the genome of culture TRIP_1 encoding 2-phenanthroyl-CoA reductase proved that the earlier discovered gene cluster is indeed encoding for enzymes involved in anaerobic phenanthrene degradation (Fig. 9) (25). Under anoxic conditions, 2-phenanthroyl-CoA reductase converted the synthesized compound 2-phenanthroyl-CoA with a specific activity of 17.6 ± 0.4 nmol/min/mg with dithionite-reduced methyl viologen as the electron donor. Although seemingly low, this activity compares well with activities of 2-naphthoyl-CoA reductases of cultures N47 and NaphS2 (N47_G38220, NPH_1753, and NPH_5475), which converted 2-naphthoyl-CoA to dihydro-2-naphthoyl-CoA with specific activities of ≈22–30 nmol/min/mg (28). A slight reduction activity was also observed with sodium dithionite or NADH only, while no activity was observed with Ti(III)-citrate or NADPH as the electron donor. Thus, we speculate that the natural electron donor for 2-phenanthroyl-CoA reductase might be a specific ferredoxin with a suitably low standard redox potential. The similar enzyme 2-naphthoyl-CoA reductase was active with dithionite and Ti^III^-citrate/methyl viologen, whereas Ti(III)-citrate, NADH, or NADPH showed almost no activity (18, 20, 28).

Another difference between 2-phenanthroyl-CoA reductase and 2-naphthoyl-CoA reductase is the oxygen sensitivity, since the reaction of 2-phenanthroyl-CoA reductase is oxygen sensitive, whereas naphthoyl-CoA reductase is not (19, 20, 27, 28). Therefore, to perform the reduction assay using 2-phenanthroyl-CoA reductase, the enzyme has to be flushed with nitrogen gas. However, this could also be due to the oxygen sensitivity of the necessary electron carrier-reduced methylviologen, which reacts spontaneously with molecular oxygen.

Similar to naphthoyl-CoA reductase, the reduction reaction catalyzed by 2-phenanthroyl-CoA reductase occurred without ATP, indicating that the enzyme belongs to the ATP-independent class type III aryl-CoA reductases.

An increase of two *m*/*z* values of the reduction product dihydro-2-phenanthroyl-CoA in LC/MS and high-resolution mass spectrometry analyses confirmed the two-electron reduction of one of the phenanthrene aromatic rings. This was also supported by NMR analysis, which was performed to determine the position of the introduced saturated bond. NMR analysis and quantum chemical calculations showed that the dihydro-2-phenanthroyl-CoA produced in the reduction reaction could be 9,10-dihydro-2-phenanthroyl-CoA (isomer **3**, Fig. 9), where the saturated bond is located in the middle ring. This compound constitutes the energetically most stable of the three putative isomers. However, it is chemically well possible that the actual product was isomer **4** or **5** (Fig. 9), with a saturated bond in ring 3 of dihydro-2-phenanthroyl-CoA, which underwent isomerization changes of the saturated bond from isomer **4** or **5** to the energetically more stable isomer **3** (Fig. 9) over time. We propose that the time used for the purification and the preparation of the compound isolated from the enzyme reaction until analysis with NMR was long enough for a complete reisomerization. This was supported by the difference in the UV-vis spectrum between the enzymatically produced dihydro-2-phenanthroyl-CoA and the synthesized 9,10-dihydro-2-phenanthroyl-CoA (isomer **3**).

The gene sequence encoding 2-phenanthroyl-CoA reductase (PITCH_a10001) indicates that the enzyme belongs to the old-yellow enzyme family. These enzymes possess domains for binding FMN, FAD, and 4Fe-4S clusters (19, 36). The heterologously expressed and purified 2-phenanthroyl-CoA reductase was a monomeric enzyme, similar to 2-naphthoyl-CoA reductase. Biochemical characterization showed that 2-phenanthroyl-CoA reductase also contains one FMN, one FAD, and one 4Fe-4S cluster as cofactors, which is supported by structural modeling analysis with Alpha Fold and MM simulation similar to 2-naphthoyl-CoA (19).

**Fig 10:**
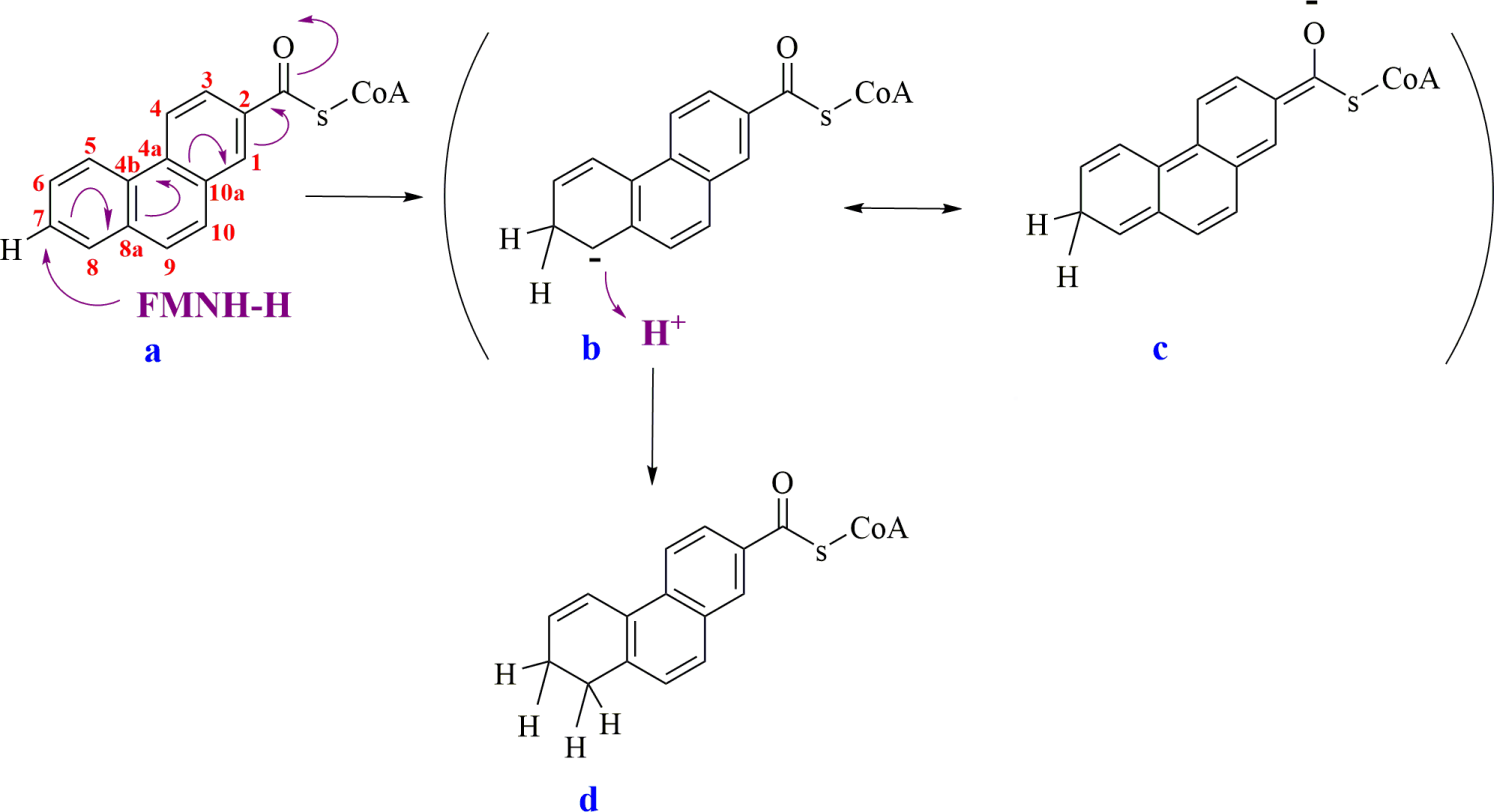
Proposed reduction mechanism of 2-phenanthroyl-CoA by 2-phenanthroyl-CoA reductase with possible resonance structure to the formation of 7,8-dihydro-2-phenanthroyl-CoA (IUPAC: 1,2-dihydro-7-phenanthroyl-CoA) (isomer **5** in Fig. 9). Compound b is a Meisenheimer complex-analogous transition state followed by stabilization by the CoA ester (compound c).

Due to the strong structural and biochemical similarities, we propose that 2-phenanthroyl-CoA reductase is following the same reduction mechanism as 2-naphthoyl-CoA reductase (18, 19). Both enzymes belong to the NADH-flavin oxidoreductase family and are proposed to facilitate the transfer of a hydride from a reduced flavin to activated alkenes that have an electron-withdrawing group. This process is followed by the protonation of the negatively charged transition state (18, 19). It is likely that after one electron reduction of the iron-sulfur cluster and FAD of 2-phenanthroyl-CoA reductase, two single electrons are transferred to FMN and, after protonation, perform a hydride transfer to the substrate, 2-phenanthroyl-CoA. The hydride could be transferred to carbon 7 of 2-phenanthroyl-CoA (**compound a**), producing a transition state analogous to a Meisenheimer complex (**compounds b and c**), followed by a protonation at carbon eight to form isomer **5** (7,8-dihydro-2-phenanthroyl-CoA) (IUPAC: 1,2-dihydro-7-phenanthroyl-CoA) (**compound d**, Fig. 10).

### Conclusion

In this study, we confirmed the reduction of 2-phenanthroyl-CoA catalyzed by 2-phenanthroyl-CoA reductase. The most likely products were the two possible isomers, 7,8-dihydro-2-phenanthroyl-CoA (IUPAC: 1,2-dihydro-7-phenanthroyl-CoA) or 5,6-dihydro-2-phenanthroyl-CoA (IUPAC: 3,4-dihydro-7-phenanthroyl-CoA). The elucidation of 2-phenanthroyl-CoA reductase shows that the anaerobic degradation of bigger PAHs with three or more rings follows a similar strategy as shown for the smaller two-ring PAH naphthalene and that the first ring reductions employ ATP-independent type III aryl-CoA reductases of the old-yellow enzyme family.

## MATERIALS AND METHODS

### Cloning of 2-phenanthroyl-CoA reductase

The gene sequence of PITCH_a10001 was recently identified among NADH-flavin oxidoreductase gene clusters from TRIP_1 culture (25). The sequence of the gene, PITCH_a10001, was codon-optimized at Eurofins Genomics, Ebersberg, Germany (Fig. S1) and amplified with PCR (forward primer, AGCGCGTCTCCAATGAAACTTTTCGAGCCGATCAAG; reverse primer, AGCGCGTCTCCTCCCGTGCAGAGCGGCAAGCA). The PCR reaction was performed with a 2× KAPA HiFi HotStart ready mix (Fisher Scientific GmbH, Schwerte, Germany) following the manufacturer’s protocol. PCR products were purified using the Monarch PCR and DNA Cleanup kits (New England Biolabs GmbH, Frankfurt, Germany). The purified PCR product was integrated into the expression plasmid, pASG-IBA103, by mixing the following reagents: pASG-IBA103 (5 ng), PCR product (2 nM), 250 mM dithiothreitol/12.5 mM ATP mix (1 µL), T4 DNA ligase (1 U), rCutSmart buffer (2.5 µL), and Esp3I restriction enzyme (5 U). The reaction was incubated for 1 h at 30°C and then transformed into NEB 5-alpha competent *E. coli* cells. The recombinant plasmid was isolated from NEB 5-alpha and transformed into BL21(DE3) competent *E. coli* cells. The recombinant plasmid was isolated from BL21(DE3) *E. coli* cells after overnight culture and delivered for Sanger sequencing (Eurofins Genomics) to confirm the successful cloning.

### Heterologous expression and purification of 2-phenanthroyl-CoA reductase

Expression of 2-phenanthroyl-CoA reductase was performed with BL21(DE3) *E. coli* cells grown in lysogeny broth medium amended with 100 µg/mL ampicillin and **≈**0.3 g of riboflavin. The culture was incubated at 37°C, followed by shaking at 200 rpm until the optical density at 600 nm reached 0.5–0.6. Then, anhydrotetracycline (0.2 µg/mL) was added to the medium as an inducer of protein expression, followed by incubation for 20 h at 20°C and 130 rpm. Cells were harvested by centrifugation at 3,100 × *g* and resuspended in 50 mM HEPES buffer, pH 8 (2 mL buffer/g wet weight *E. coli* cells). Subsequently, the resuspended cells were disrupted using a French pressure cell at 82.7 bar, and the supernatant was collected by centrifugation for 1 h at 16,000 × *g*. 2-Phenanthroyl-CoA reductase was purified by subjecting the supernatant to gravity flow using Strep-Tactin XT 4Flow High Capacity (IBA, Göttingen, Germany) according to the manufacturer’s instructions. Elution of the target protein from the column was performed using a 50 mM biotin solution, and the obtained purified enzyme was concentrated using a 50 kDa-cut-off Pierce Protein Concentrator (Thermo Fisher Scientific, Waltham, USA). The concentration of purified 2-phenanthroyl-CoA reductase was determined by Bradford assay (37). SDS-PAGE was used to determine the purity and molecular mass of 2-phenanthroyl-CoA reductase. 12% Mini-PROTEAN TGX precast gel cassette (Bio-Rad, Hercules, USA) was used for SDS-PAGE analysis and stained with ROTI Blue colloidal coomassie stain (Roth, Karlsruhe, Germany), and the purified enzyme’s molecular weight was determined by PageRuler prestained protein ladder (Thermo Fisher Scientific).

### Synthesis and analysis of 2-phenanthroyl-CoA

2-Phenanthroyl-CoA ester was synthesized by reacting carbonyldiimidazole (4.2 mg) with 2-phenanthroic acid (32 µM) in 200 µL tetrahydrofuran (THF) for 2 h at 900 rpm (ThermoMixer C, Eppendorf, Germany) and at 22°C in a nitrogen-filled glove box. Then, CoA-ester (5 mg) was dissolved in 250 µL sodium bicarbonate (100 mM), added to the THF solution, and incubated for 4 h. After adding 20% formic acid (10 µL), 2-phenanthroyl-CoA ester was extracted with 100 µL ethyl acetate. The obtained 2-phenanthroyl-CoA ester was frozen at −70°C and subsequently freeze-dried overnight. The dried 2-phenanthroyl-CoA ester was dissolved in 2 mL aqueous 0.1% (vol/vol) formic acid and purified using an SPE column (CHROMABOND C18 Hydra, 45 µm, 6 mL; MACHEREY-NAGEL GmbH, Düren, Germany). The column was activated with 2 mL acetonitrile and equilibrated with the same amount of aqueous 0.1% (vol/vol) formic acid. The dissolved product was loaded on the column and washed twice with 2 mL of aqueous 0.1% (vol/vol) formic acid. The column was developed in steps of 4 mL acetonitrile (5%, 10%, 20%, 40%, 60%, and 100%) in aqueous 0.1% (vol/vol) formic acid. The successful synthesis of 2-phenanthroyl-CoA ester was checked by LC-MS, and the fraction with the highest concentration of the purified substrate was freeze-dried and stored at −20°C.

### Determination of 2-phenanthroyl-CoA reductase activity

The purified 2-phenanthroyl-CoA reductase was used to detect the enzymatic activity under anoxic conditions in triplicate. The reduction assay (200 µL) contained 100 µL of the concentrated, purified 2-phenanthroyl-CoA reductase (50–100 µg) and 100 µL of a mixture of 1 mM NADH, 50 µM FMN, 1 mM FAD, and 100 µM 2-phenanthroyl-CoA ester dissolved in 50 mM HEPES buffer at pH 7.5. Sodium dithionite (5 mM), Ti(III)-citrate (5 mM), NADPH (5 mM), as well as methyl viologen (1 mM) reduced with dithionite (1 mM) were tested as electron donors. The reaction was performed with or without ATP to test ATP dependence. The reaction was started by adding the enzyme solution to the reaction mixture and incubating at 30°C and 900 rpm in a Thermomix Block (ThermoMixer C, Eppendorf) inside a glove box with nitrogen atmosphere. The reaction was stopped after 0, 45, and 90 min by taking an aliquot (40 µL) and adding a double volume of methanol. Subsequently, all reaction tubes were centrifuged at 16,000 × *g* for 60 min, and the supernatants were analyzed with LC/MS for 2-phenanthroyl-CoA and dihydro-2-phenanthroyl-CoA.

### Conversion of dihydro-2-phenanthroyl-CoA to the free acid dihydro-2-phenanthroic acid

After stopping the enzymatic reaction described in the previous section in a double volume of methanol, the methanol was evaporated using SpeedDry Vacuum Concentrator (Martin Christ Gefriertrocknungsanlagen GmbH, Osterode am Harz, Germany). The product was hydrolyzed at room temperature using 5 M KOH at pH 13 then titrated with HCl at pH 1. The titrated product was left stirring for 30 min at room temperature and then extracted three times with chloroform. The free dihydro-2-phenanthroic acid stayed in the lower phase in the chloroform. The lower phase was collected, and the extraction process was repeated for two more times. The collected lower phase was dried over Na_2_SO_4_ to remove remaining water from the organic phase then filtered using a 0.45 µm pore-sized CHROMAFIL filter (MACHEREY-NAGEL GmbH). The organic solvent was evaporated using the SpeedDry Vacuum Concentrator, and the crude product was purified using an SPE column (CHROMABOND C18 Hydra, 45 µm, 6 mL; MACHEREY-NAGEL GmbH). The column was activated with 2 mL acetonitrile and equilibrated with the same amount of 25 mM potassium phosphate buffer at pH 6.8. The crude product was loaded on the column, and the free acid was eluted with steps of 4 mL acetonitrile (5%, 10%, 20%, 40%, 60%, and 100%) in a 25 mM potassium phosphate buffer at pH 6.8. The successful production and purification of dihydro-2-phenanthroic acid were checked by LC-MS, and the fraction with the highest concentration of the purified substrate was freeze-dried and stored at −20°C. The free dihydro-2-phenanthroic acid was obtained as a white solid. The method of acid production and purification was adapted from reference 18.

### LC-MS analysis

LC/MS analyses were performed using a Shimadzu LC-2040C system coupled to a LC-MS-2020 single quadrupole mass-spectrometer (Shimadzu Deutschland, Duisburg, Germany). Metabolites were separated on a Nucleodur C18 Gravity-SB column (MACHEREY-NAGEL GmbH) with 250 mm column length, 4.6 mm column inner diameter, and 5 µm particle size, at a column oven temperature of 35°C. A linear gradient was run from 90% solvent A (0.1% [wt/vol] ammonium formate) and 10% solvent B (acetonitrile) to 90% solvent B for 30 min with a flow rate of 0.4 mL/min. The acquisition time started from 3 min and ended after 18 min. The analytes were detected in positive as well as negative ionization modes using an electrospray ionization system. The ion spray voltage of the ESI system was adjusted to 4,500 V in the positive mode and −4,500 V in the negative mode at 350°C. In addition to a specific search for certain molecular masses of putative target metabolites, mass-over-charge ranges from 50 to 1,050 *m*/*z* were scanned.

### Quadrupole time-of-flight/LC-MS analysis

The LC-MS system included a 1290 Infinity II Multisampler (G7167B), a 1290 High-Speed Pump (G7120A), a 1920 MCT (G/116B), and a 1290 DAD FS (G7117A), all connected to a 6546 LC/Q-TOF with a DUAL-AJS ESI source from Agilent Technologies, USA. For the LC-MS analyses, a mixture of water (A) and methanol (B), each containing 0.1% (vol/vol) formic acid, was utilized. The quadrupole time-of-flight was operated in full-scan, data-dependent MS/MS acquisition mode, covering a mass range from 100 to 1,100 Da in both positive and negative ion modes. Each measurement was conducted in triplicate to ensure accuracy and reproducibility. The ion source operational parameters were set as follows: gas temperature at 320°C, drying gas flow at 8 L/min, nebulizer pressure at 2.4 bar, sheath gas temperature at 350°C, sheath gas flow at 11 L/min, and a spray voltage of 3,500 V.

### 2-Phenanthroyl-CoA reductase characterization using UV/vis spectroscopy

UV/vis analysis was used to examine if the enzyme is expressed and isolated in the oxidized or reduced form. Subsequently, 20 µM purified enzyme was stepwise reduced with sodium dithionite (0.05 mM) in gas-tight, nitrogenflushed quartz cuvettes, and the spectra were taken with an Infinite M200 Pro TECAN Spectrophotometer (Tecan Group Ltd., Switzerland). Moreover, solutions of coenzyme A, 2-phenanthroyl-CoA, the synthesized 9,10-dihydro-2-phenanthroyl-CoA, and the final reduction product, dihydro-2-phenanthroyl-CoA, were separately analyzed with UV/vis.

### Determination of flavin and iron content

The flavin content of 2-phenanthroyl-CoA reductase was measured by LC-MS, as described (20). Briefly, 0.5 H_2_SO_4_ (10 µL) was added to 200 µL HEPES buffer (50 mM, pH 7.8) containing the purified enzyme (35 µg), and the reaction was incubated in the dark in an ice bath for 15 min followed by 10 min centrifugation at 16,000 × *g* at 4°C. The supernatant was collected, and the pellet was resuspended in 30 µL H_2_SO_4_ (0.02 M in 50 mM HEPES buffer, pH 7.8) and incubated again in the dark in the ice bath for 15 min followed by 10 min centrifugation at 16,000 × *g* at 4°C. The supernatants were combined, and 100 µL was used for LC-MS analysis. The iron content was measured using a colorimetric method developed from reference 38.

### 2-Phenanthroyl-CoA reductase kinetic properties

Specific activity was determined based on the substrate conversion to dihydro-2-phenanthroyl-CoA in micromolar (monitored by LC-MS) per milligram of protein within the first 10 min. Specific activity was calculated from an average of three independent replicates. The 2-phenanthroyl-CoA reductase enzyme assay was performed with different initial concentrations of 2-phenanthroyl-CoA (2–100 µM) to determine the initial reaction rate, the *K*_*m*_, and the *V*_max_.

### NMR analysis

NMR experiments were performed on Avance HD spectrometers (Bruker BioSpin GmbH & Co. KG, Ettlingen, Germany) operating at ^1^H Larmor frequencies of 600.13 and 700.22 MHz, respectively. While the first one was equipped with a cryogenic, nitrogen-cooled BBO probe (“Prodigy”), the latter one worked with a helium-cooled cryoprobe (TCI). For structural elucidation and signal assignment, one-dimensional ^1^H and ^13^C spectra, together with the two-dimensional ^1^H COSY, ^1^H ROESY, ^1^H-^13^C HSQC, and ^1^H-^13^C HMBC spectra, were recorded using the Bruker pulse sequence library associated with the TOPSPIN (versions 3.7 and 4.1) package. Chemical shifts are reported with respect to tetramethylsilane (0.0 ppm for ^1^H and ^13^C NMR) using the ^1^H resonance of the solvent as secondary reference. For all NMR experiments, the temperature of the sample was set to 298 K.

### Quantum chemical calculations

Theoretical calculations were carried out with the Gaussian 16 program package to estimate the stability of the three possible isomers of the reaction product dihydro-2-phenanthroyl-CoA (39). The geometrical parameters of the reactants and products were optimized by the B3LYP method (40) with the 6–311 + G(d,p) basis set (41, 42). To account for solvent effects, the polarized continuum model was applied, with methanol as the solvent (43–45). All molecules exhibited C1 symmetry. Frequency calculations were performed for all the structures studied to confirm the absence of imaginary frequencies.

### Sequence alignment with 2-naphthoyl-CoA reductase

The protein sequences of the genes PITCH_a10001 and N47_G38220 were downloaded from the UniProt database (46). The alignment was performed with the Biotite python package. A BLOSUM62 matrix with gap penalties of −10 and −1 and without terminal gap penalty was used for the alignment (47).

### Modeling of 2-phenanthroyl-CoA reductase and cofactors

The predicted structure of 2-phenanthroyl-CoA reductase was downloaded from the Alpha Fold Protein Structure Data Base (32). To populate the structure with the cofactors FMN, FAD, and the [4Fe-4S] cluster, AlphaFill was used with its *YASARA* optimization function to transfer the cofactors from the protein structure 6QKG (29). The structure was aligned to 6QKX, and the metabolite 2-naphthoyl-CoA was transferred from 6QKX to the model with pyMol to model the substrate 2-phenanthroyl-CoA into the structure. Then, Avogadro was used to build the remaining ring. The structure of the ligand was optimized with the AutoOptimization function using the UFF forcefield (48, 49).

### MM simulation

The PDBfixer package was used to remove heteroatoms and to prepare the protein for MM simulation, to add non-terminal missing residues, and to add missing hydrogens (50). The ligand preparation was done with the rdkit package. The ligands were extracted from the PDB; their bond order was fixed via the AssignBondOrdersFromTemplate function; and missing hydrogens were added with the AddHs function (51). Afterward, an openff interchange was used to parametrize the molecules and create an interchange object (52). The force fields used to create this interchange were the sage 2.2.0 force field and the ff14sb force field (53, 54). The final MM simulation was done using openmm, with a Langevin integrator at 303.15 K, for 1 ps and in 2 fs steps (55). To validate the simulation result, the protein ligand interactions were analyzed with the PLIP tool (34, 35).

## Data Availability

The data supporting the findings of this study can be found in the supplemental material.

## SUPPLEMENTAL MATERIAL

Supplemental material aem.00166-25-s0001.docx Figures S1 to S9, Table S1, and supplemental methods.

ASM does not own the copyrights to Supplemental Material that may be linked to, or accessed through, an article. The authors have granted ASM a non-exclusive, world-wide license to publish the Supplemental Material files. Please contact the corresponding author directly for reuse.
