# Supplementary material for: Characterization of 2-phenanthroyl-CoA reductase, an ATP-independent type III aryl-CoA reductase involved in anaerobic phenanthrene degradation
Source: Appl Environ Microbiol. 2025 Apr 17;91(5):e00166-25. doi: 10.1128/aem.00166-25 (PMC12093976; doi:10.1128/aem.00166-25)
Supplement: Supplemental material — Figures S1 to S9, Table S1, and supplemental methods. [file aem.00166-25-s0001.docx]

**Characterization of 2-phenanthroyl-CoA-reductase, an ATP-independent type III aryl-CoA reductase involved in anaerobic phenanthrene degradation**

Nadia A. Samak^1*^, Frederik Götz^1^, Khadija Adjir^2^, Torsten Schaller^3^, Marvin Häßler^4^, Oliver J. Schmitz^4^, Jonas Fax^3^, Gebhard Haberhauer^3^, Alina Surmeneva^1^, Rainer U. Meckenstock^1*^

^1^ Environmental Microbiology and Biotechnology (EMB), Faculty of Chemistry, University of Duisburg-Essen, Universitätsstr. 5, 45141 Essen, Germany

^2^ Laboratory of Thermodynamics and Molecular Modeling, Faculty of Chemistry, USTHB, BP32 El Alia, 16111 Bab Ezzouar, Algiers, Algeria

^3^ Organic Chemistry, Faculty of Chemistry, University of Duisburg-Essen, Universitätsstr. 5, 45141 Essen, Germany

^4^ Applied Analytical Chemistry, Faculty of Chemistry, University of Duisburg-Essen, Universitätsstr. 5, 45141 Essen, Germany

^*^ Corresponding author, E-mail:

[nadia.samak@uni-due.de](mailto:nadia.samak@uni-due.de); Tel. +49 (0)201 183-7089

[rainer.meckenstock@uni-due.de](mailto:rainer.meckenstock@uni-due.de); Tel. +49 (0)201 183-6601; Fax +49 (0)201 183-6603

**Supplementary Material**

ATGAAACTTTTCGAGCCGATCAAGATCGGGAAAGTTAGTGTCAAAAACCGGATTGTTATGGCCCCAATGACGAATCACTTTGCAGACAAAGGCTTTGTCACCGAACGTATGGTGTCGTTCTATGAAGCCCGCGCTCGGGGTGGCAGTGGTCTGATTACGATCGAAGATGCCATTGTGGACTATCCGATTGGGAACAATACCGCCAATCCGTTAGCGATTGACCACGAAAAGTACATCCCGATGTTGAAGAAACTGTCATCGACCATTAAATCCCATGGTTGCGTACCGATGGTTCAGCTGAGTCACGCAGGACGTCGTGCGGGACGTGTAAACCCGGATACAGGCTGCATTGAAACGACCCAACATCGTCTGCCAGTAGCCCCGAGTGCCTTAGCACATCCGTTTCCTGGGCATGTGGTACCGCGTGTTCTGCGCGTTGAAGAGATCGAAGCGATTACCGAAAAATTTGTGCAAGGTGCACGTCGTGCGGTGGAAGCGGGCTTTGACATCGTGGGCTTACACTGTGCTCACATGTATCTGATCGGCCAGTTCCTGTCACCATGGGCGAATAAACGCATGGATGACTATGGCGGCACTCTGGAAAATCGCATGCGCTTCATTATCAACATCATTCGCCGCATCAAACGGGAGATTGGGGAGGATTTTCCGTTGGTGTGTCGTATGAATGGTGCCGAACCTGAAGGCGGCAATACCTTGCGCGAGATCCAAGACATCGCAGTCAAACTGCAAAGCGCGGGCATTAACGCTCTGCATGTCTCTGTGGGTTTTGGTCCAGTCTTATGGGAGAAAGGGTTCATGCCTGCGGAAGCGCCGATCGGAATGCCGGAGGGTTGCATCGTTAATCTGGCGGAGAATATTAAACGCGTGGTTACAATTCCGGTTATTACGGTGAACAAGATTCGTCATGTCGACTTTGCCGAGAATATTCTGCAGCGTAAACGCGCGGATATGATCGCATTGGGTCGTGCGTTGTTGGCTGATCCGATGTGGCCGTATAAAGCGATGAACAATAAAGCCGCAGAAATTCGCCCGTGTGTCTCGTGCTGTCAAGGATGCGTCAAAAACATCGAAACCGGCAATCCAATCAGCTGCCTTGCAAATCCGCTGGTTGGGCGCGAATACGAAATCATGCTCGATCGTGTACCGCCGGATAGCGTTAAAAAGATTCTGGTGATTGGCGGCGGTCCGGCAGGTTTAATGACGGCCATTATCGCCGCGAAACGGGGACACAAGGTGTCTATTTGGGAAAAGGAGAACCGCCTGGGTGGTGAGATGCATCTGGCGATGATGCCGCCTCGCAAACAGGAATTTCGGGAATTAATGGAGTACTTAATCTTTCGCGTGAATAGCCTCGGCATTGAAGTGAAACTCAACACTCTTGCTGGTGCTCGTACGGTTGGTCACTTCGATGCAGATGCCGTGGTGGTTGCCATTGGAAGCGAAACCATGATGCCGACGATTGCTGGCATCGAGAATTCGAACGTAATCAGCGCGATCAAAGCGTTTGAAGATGAGGCGAATGTCGGTGCGAAAGTGGTCATTATTGGCGGCGGCCTGATTGGGTTGGAGGCGGCAGAAAGCTTCAGCCAGAAAGGCAAAGCCATCACCATCGTTGAGATCAAAGAGGATGTTGGCTCTAACATGCCAATGCTCGTGAAGATTCCTCTGCTGATCACCTTACAGGAACGTGGCGTGAACATTCTGACAGGGTCATCCGTACGCAAGATCGATTTGACTGGTGTGGAAGTAGAGCATGGCGGCACCGTGAAATTCCTGGATTGTGAAACCGTGATTATTGCATCTGGTGGCAAATCCAATCATAGTCTCGAAGAACAGATCCGCTCTGCCGGTAAAAACGTGTATTCCGTGGGGTCGTCGAATCTTCTCGGGGATATGCTTGCCGCTCTGCACAAT

**Fig. S1.** PITCH_a10001 codon optimized gene sequence (1982 bp)


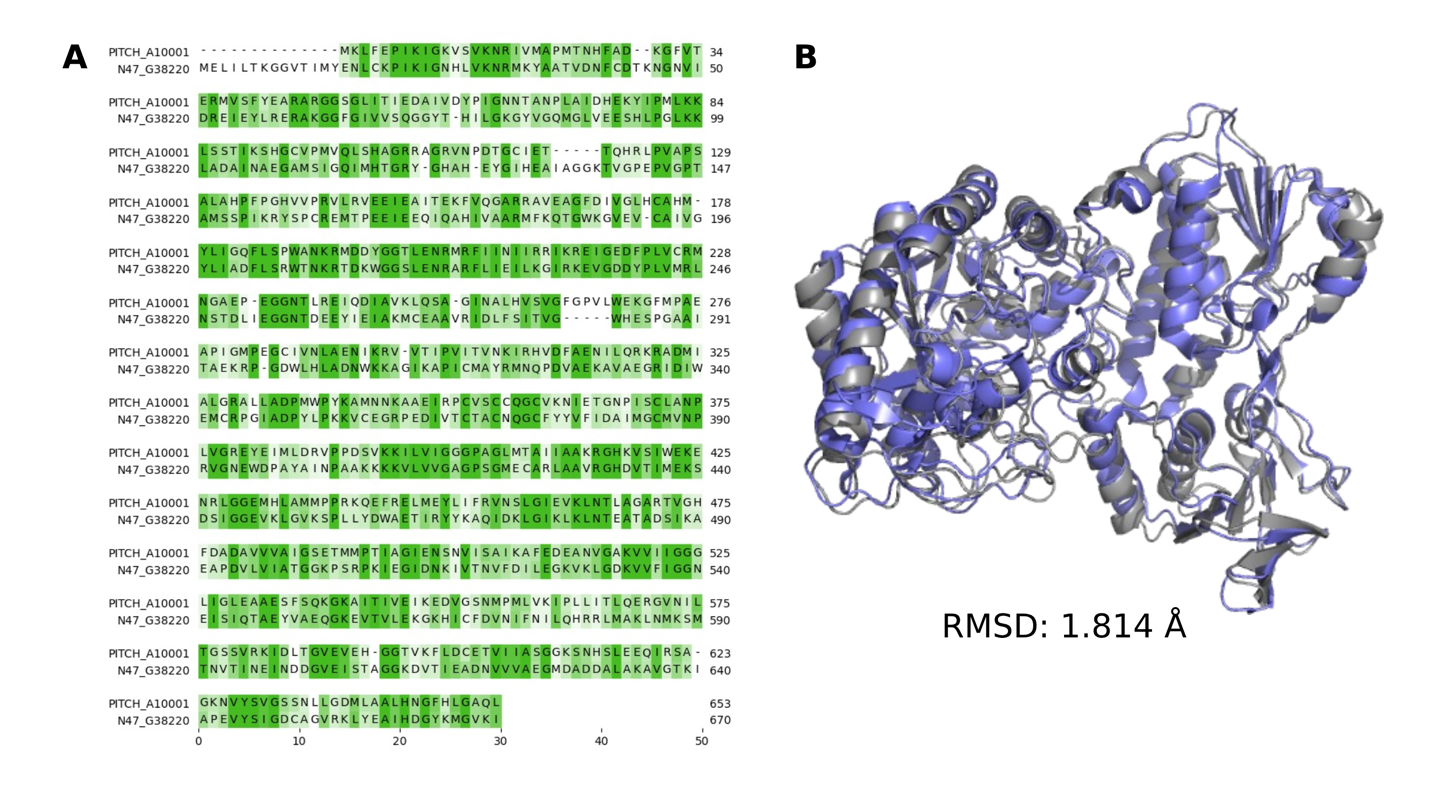


**Fig. S2.** Sequence alignment (**A**) and homology fitting (**B**) between 2-phenanthroyl-CoA- reductase (PITCH_A10001) and 2-naphthoyl-CoA reductase (N47_G38220).


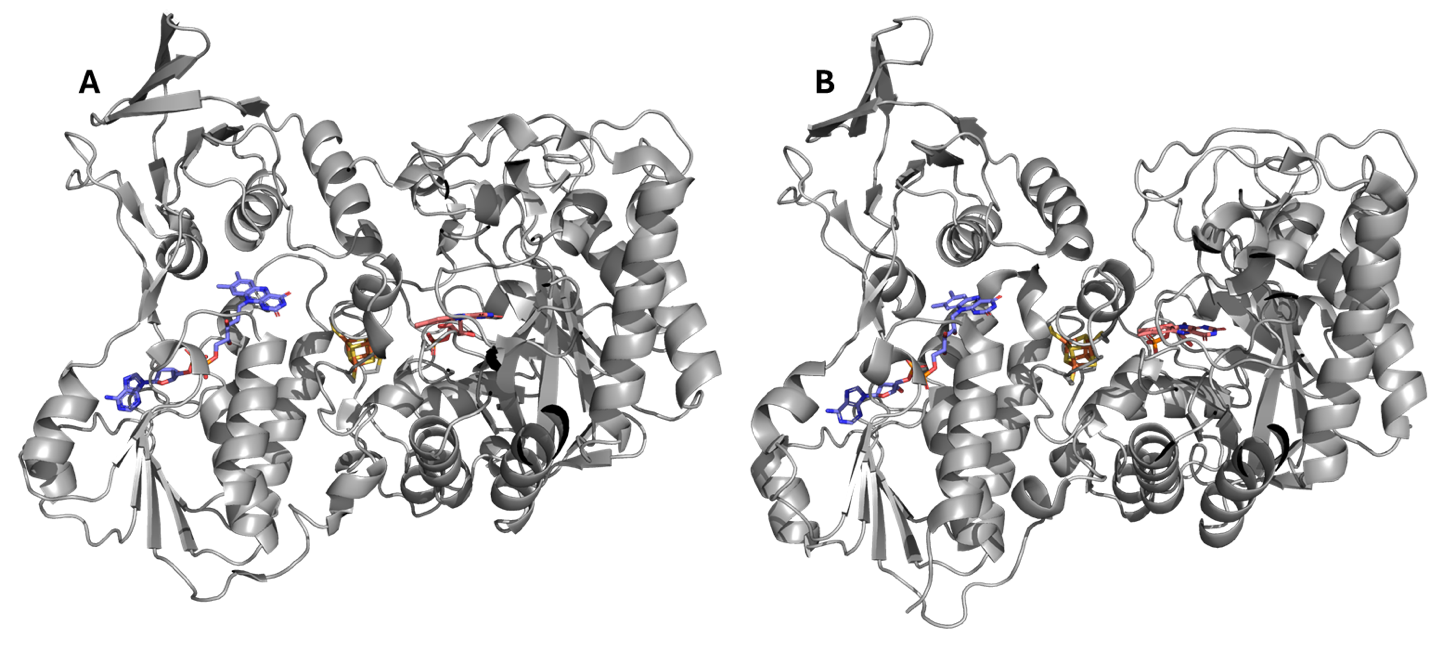


**Fig. S3.** Comparison of the AlphaFold generated structure of (**A)** 2-phenanthroyl-CoA-reductase and (**B)** 2-naphthoyl-CoA reductase (PDB: 6QKG). FMN is shown in blue and FAD in salmon.


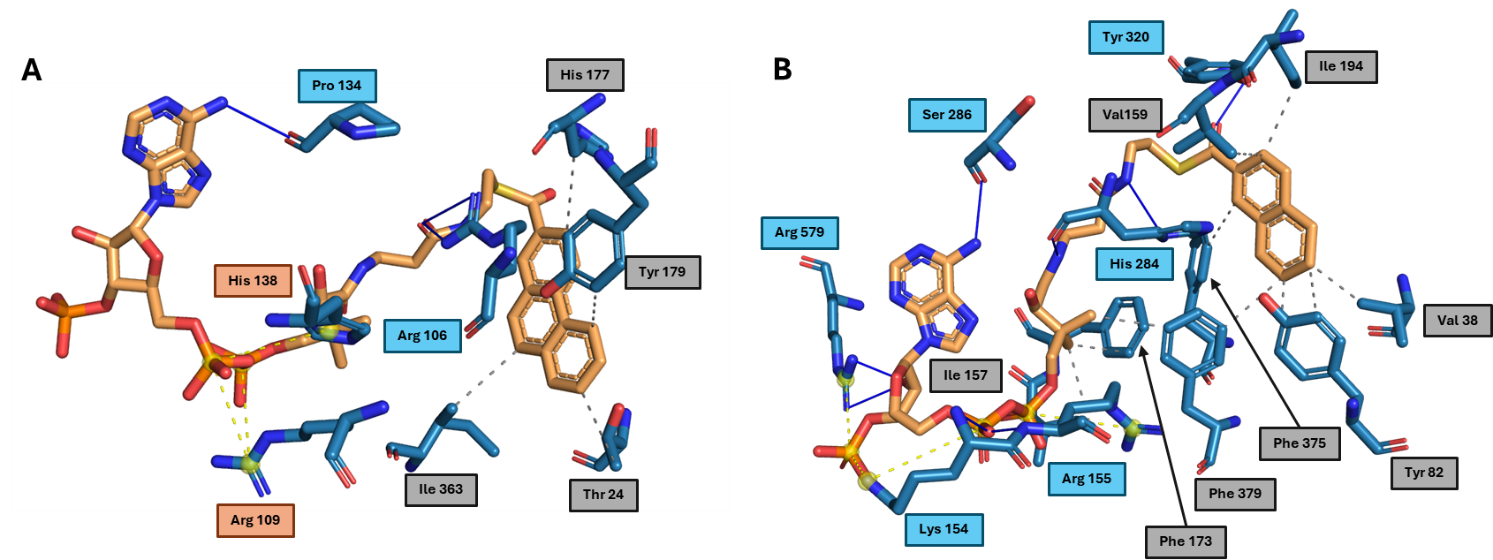


**Fig. S4.** PLIP of 2-phenanthroyl-CoA/2-naphthoyl-CoA of (**A)** 2-phenanthroyl-CoA-reductase and (**B)** 2-naphthoyl-CoA reductase. Blue solid line show hydrogen bonds, grey dotted lines hydrophobic interactions, and yellow/orange dotted lines salt bridges. The labels of the interacting amino acid sidechains are coloured in the corresponding colour (1, 2).

**
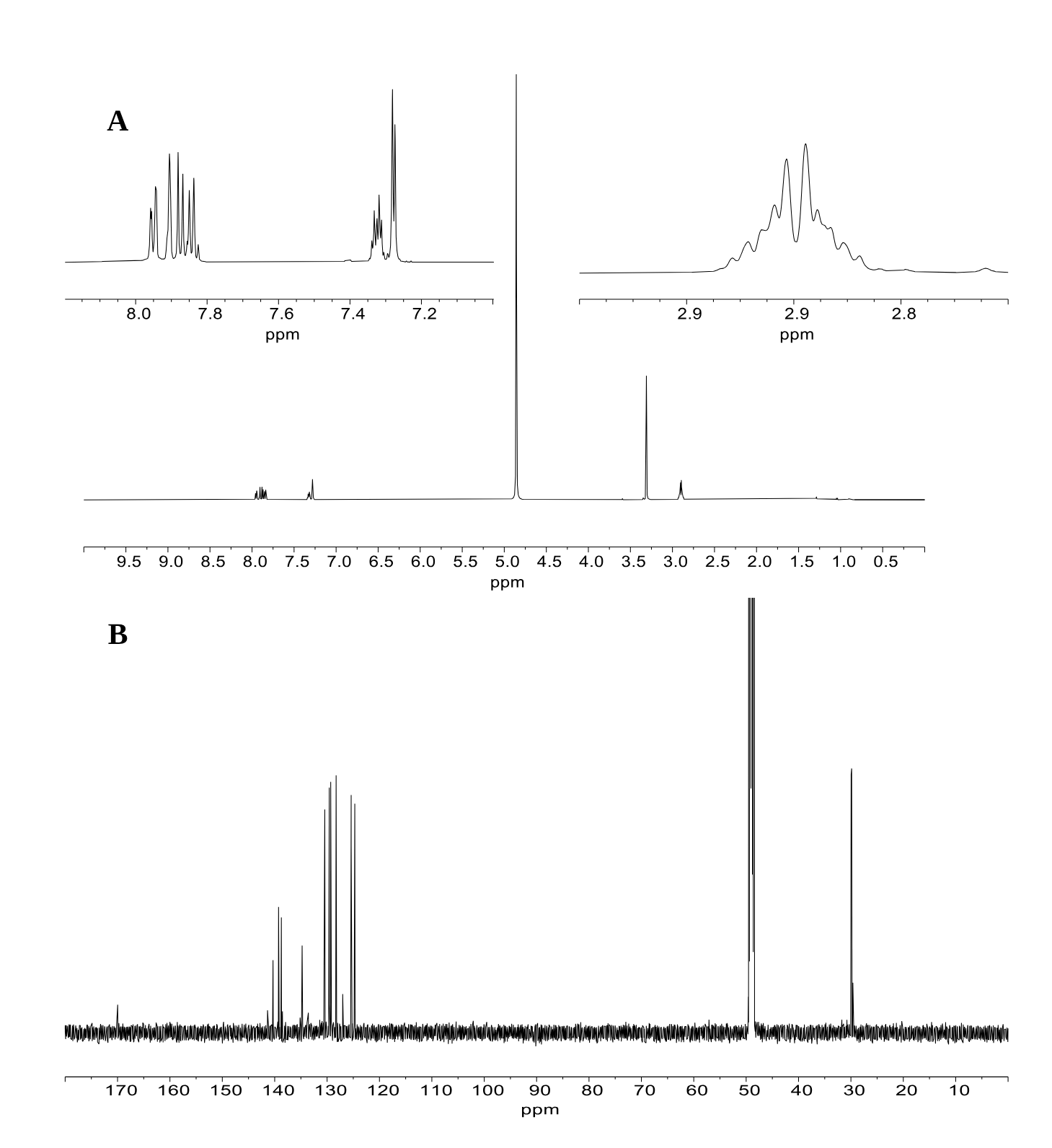
**

**Fig. S5.** 600 MHz ^1^H NMR spectrum **(A)** of synthetic 9,10-dihydrophenanthrene-2-carboxylic acid in d4-methanol. Enlarged on top: resonances of the protons on the aromatic rings (7.2 to 8 ppm) and the methylene protons (2.9 ppm). 150 MHz ^13^C NMR spectrum **(B)** of synthetic 9,10-dihydrophenanthrene-2-carboxylic acid in d4-methanol.

**
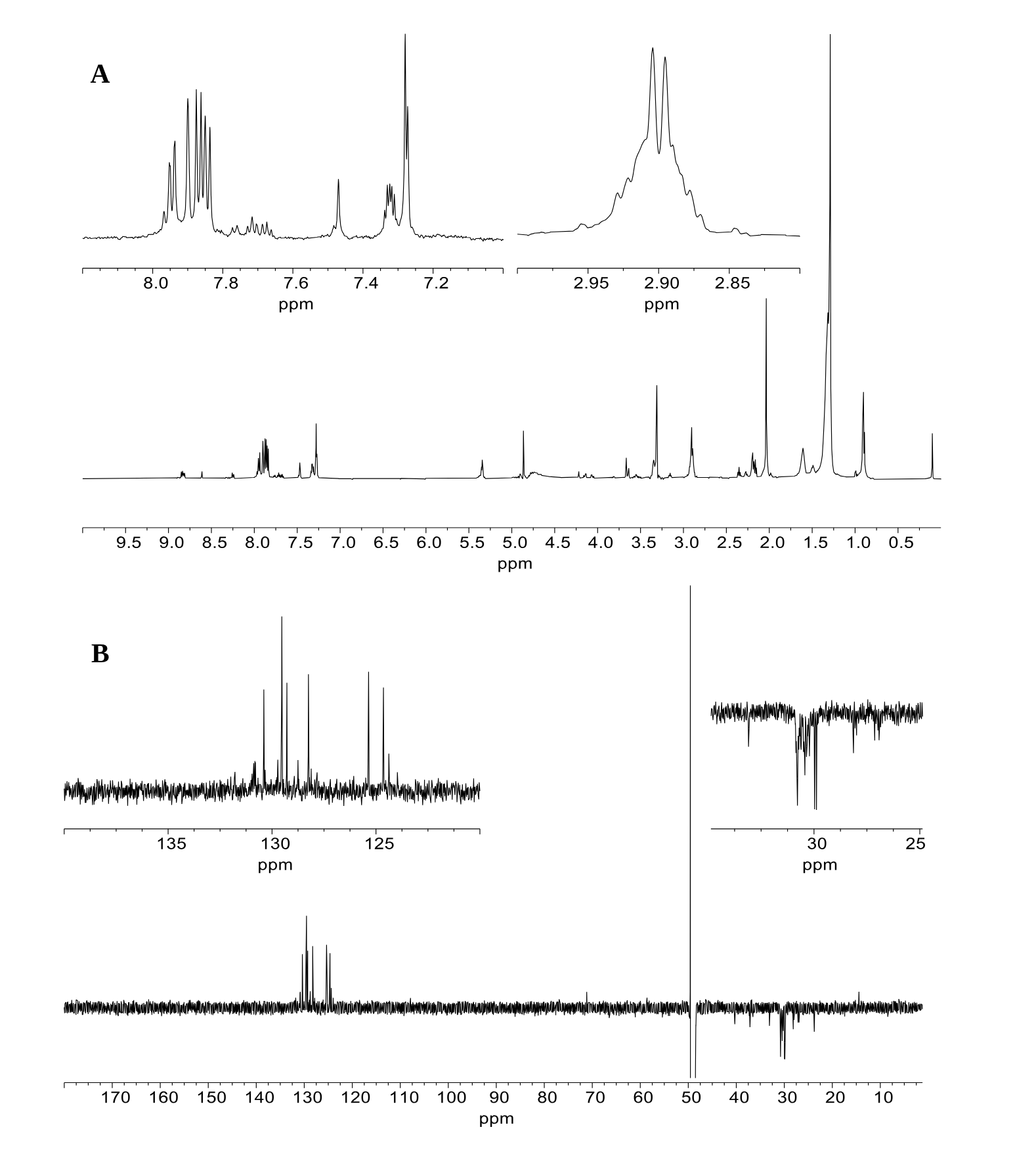
**

**Fig. S6.** 600 MHz ^1^H NMR spectrum **(A)** of enzymatically produced dihydrophenanthrene-2-carboxylic acid in d4-methanol. Enlarged on top: resonances of the protons on the aromatic rings (7.2 to 8 ppm) and the methylene protons (2.9 ppm): The spectrum was recorded with suppression of the HDO signal of methanol (4.79 ppm). 150 MHz ^13^C NMR spectrum **(B)** of enzymatically produced dihydrophenanthrene-2-carboxylic acid in d4-methanol. Enlarged on top: resonances of the carbon atoms of the aromatic rings (124 to 132 ppm) and the methylene carbons (30 ppm). The spectrum was recorded with a DEPT_Q sequence resulting in negative intensities for signals of quarternary and methylene carbons. Due to the low concentration of the sample the resonances of quarternary carbons could not be detected.

| Position | ^1^H (ppm) | ^13^C (ppm) |
| --- | --- | --- |
| 1 | 7.90 | 130.4 |
| 2 |  | 131.2 |
| 3 | 7.95 | 129.5 |
| 4 | 7.87 | 124.6 |
| 4a |  | 140.1 |
| 4b |  | 138.6 |
| 5 | 7.84 | 125.3 |
| 6 | 7.32 | 128.3 |
| 7 | 7.27 | 129.5 |
| 8 | 7.28 | 129.3 |
| 8a |  | 139.2 |
| 9 | 2.89 | 29.9 |
| 10 | 2.90 | 30.0 |
| 10a |  | 134.8 |
| 11 |  | 170.5 |

**Table S1.** ^1^H and ^13^C chemical shifts of 9,10-dihydrophenanthrene-2-carboxylic acid in d4-Methanol.

**
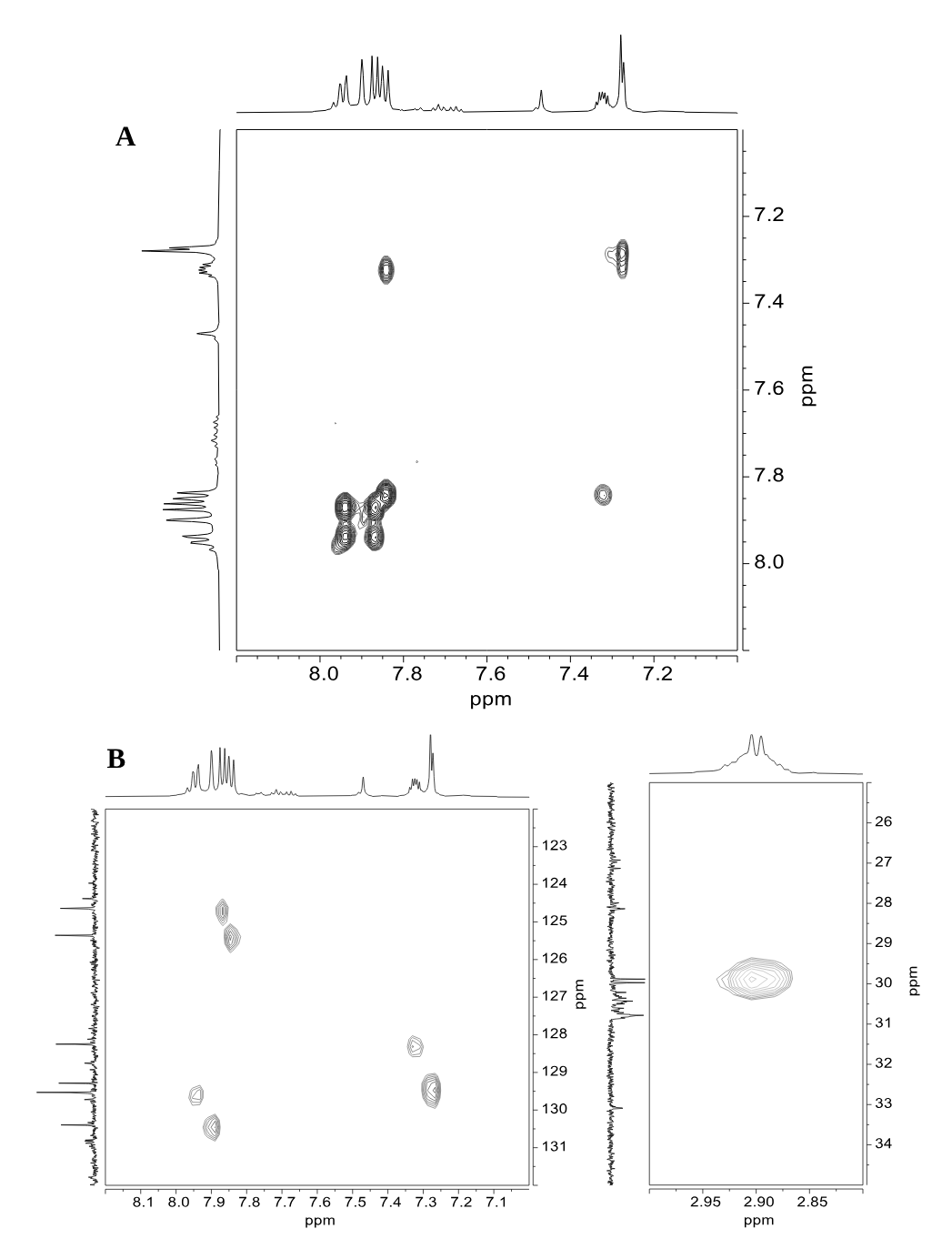
**

**Fig. S7.** 600 MHz ^1^H COSY spectrum **(A)** of enzymatically produced dihydrophenanthrene-2-carboxylic acid in d4-methanol. Only the resonances of the protons on the aromatic rings (7.2 to 8 ppm) is displayed. No correlation to signals of methylene protons have been observed. 600 MHz ^1^H-^13^C HSQC spectrum **(B)** of enzymatically produced dihydrophenanthrene-2-carboxylic acid in d4-methanol split into the spectral regions of the aromatic rings and of the methylene groups, respectively.


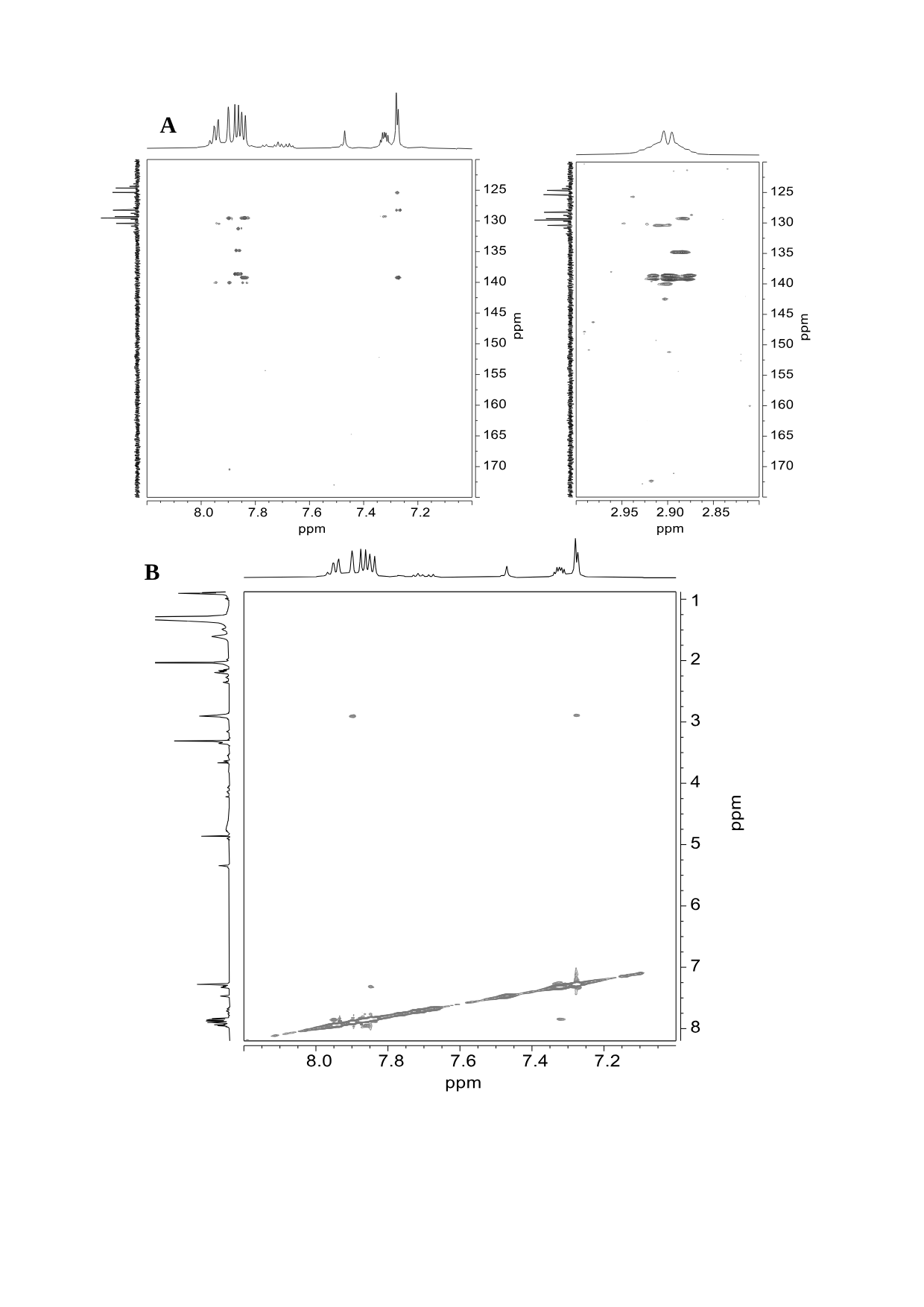


**Fig. S8.** 600 MHz ^1^H-^13^C HMBC spectrum (A) of enzymatically produced dihydrophenanthrene-2-carboxylic acid in d4-methanol splitted into the spectral regions of the aromatic rings and of the methylene groups, respectively. Here, also the correlation to signals of the quarternary carbons (which could not be detected directly) were observed allowing the full assignment of all resonances. 600 MHz ^1^H NOESY spectrum (B) of enzymatically produced dihydrophenanthrene-2-carboxylic acid in d4-methanol. Two correlation peaks for the methylene protons were detected proving the position of these groups at the middle ring.

**Fig. S9.** UV/vis spectra of the reduction assay showing the peak position difference between the enzymatically reduced product dihydro-2-phenanthroyl-CoA (with unknown isomer), the 2-phenanthroyl-CoA (substrate), and the CoA ester (control). The UV/vis spectra also show the spectral difference between the enzymatically reduced product and the chemically synthesized 9,10-dihydro-2-phenanthroyl-CoA (isomer 3, **Fig. 9**), confirming that they are two different isomers.

**Chemical synthesis of 9,10-dihydro-2-phenanthroyl-CoA (isomer 3)**

All chemicals were of reagent grade and were used as purchased from Alfa Aesar, Acros Organics, TCI or Sigma-Aldrich without further purification. Reactions were monitored by TLC analysis on Macherey-Nagel silica gel 60 F254 thin layer plates. Column chromatography was carried out on Macherey-Nagel silica 60 (40−63 µm). ^1^H and ^13^C NMR spectra were measured with a Bruker Avance HD 600 spectrometer. Chemical shifts δ are given in ppm. The spectra are referenced to the peak of the protium impurity of the deuterated solvent (CD_3_OD, ^1^H: 3.31 ppm, ^13^C: 49.00 ppm). Signal multiplicities in ^1^H NMR spectra are referred to as s (singlet), d (doublet), dd (doublet of doublet) and m (multiplet). ^13^C NMR spectra were measured with ^1^H decoupling. ^13^C assignment was achieved using COSY, HSQC and HMBC spectra. The ^13^C signals are referred to as p (primary), s (secondary), t (tertiary), q (quaternary) and ar (aromatic) carbon atoms. HR-MS spectra were measured with a Bruker BioTOF III spectrometer with electrospray ionization (ESI) as the ionization method. UV/Vis absorption spectra were measured with a Jasco V-550 spectral photometer. IR absorption spectra were measured with a Shimadzu IR Tracer-100 spectral photometer. Melting points were determined in a Büchi B-540 melting point apparatus with an open capillary and are uncorrected.

**Synthesis of 2-acyl-9,10-dihydrophenanthrene.** 9,10-dihydrophenanthrene (1.10 g, 6.10 mmol, 1.00 eq) was dissolved in 10 mL dry dichloromethane in an argon atmosphere. The stirred solution was cooled in an ice bath and aluminium chloride (1.22 g, 9.15 mmol, 1.50 eq) was added in one portion, after which the solution turned brown. After 10 minutes a solution of acetyl chloride (0.52 mL, 7.32 mmol, 1.20 eq) in 3 mL dry dichloromethane was added dropwise to the reaction mixture over 5 minutes, as the color slowly changed to green. The reaction mixture was stirred in an ice bath for 30 minutes and subsequently stirred for 3 hours at room temperature. After cooling the mixture in an ice bath, brine was added under vigorous stirring. The layers were separated and the aqueous phase was extracted three times with dichloromethane. The combined organic layers were washed with brine once and dried over anhydrous magnesium sulfate. After filtration the solvent was removed *in vacuo* and the crude product purified by flash column chromatography (SiO_2_, cyclohexane/ethyl acetate 3/1), yielding 2-Acyl-9,10-dihydrophenanthrene (0.55 g, 2.48 mmol, 41%) as a slightly yellow oil. $\text{R}_{\text{f}}$ (SiO_2_, cyclohexane/ethyl acetate 3/1) = 0.43. ^1^H NMR (600 MHz, CD_3_OD): δ = 7.92–7.90 (m, 1H, C_ar_H), 7.88–7.86 (m, 2H, C_ar_H), 7.83 (d, ^3^*J*_H,H_ = 7.8 Hz, 1H, C_ar_H), 7.33–7.30 (m, 1H, C_ar_H), 7.29–7.26 (m, 2H, C_ar_H), 2.92–2.90 (m, 2H, CH_2_), 2.88–2.85 (m, 2H, CH_2_), 2.60 (s, 3H, C(O)CH_3_) ppm. ^13^C NMR (151 MHz, CD_3_OD): δ = 200.2 (q, C(O)CH_3_), 140.7 (q, C_ar_C(O)CH_3_), 139.3 (q, C_ar_), 138.9 (q, C_ar_), 137.0 (q, C_ar_), 134.6 (q, C_ar_), 129.8 (t, C_ar_H), 129.3 (t, C_ar_H), 129.2 (t, C_ar_H), 128.4 (t, C_ar_H), 128.3 (t, C_ar_H), 125.5 (t, C_ar_H), 124.9 (t, C_ar_H), 29.9 (s, CH_2_), 29.8 (s, CH_2_), 26.7 (p, C(O)CH_3_) ppm. HRMS (ESI): [C_16_H_14_O+H]^+^: calculated: 223.1117; observed: 223.1116. [C_16_H_14_O+Na]^+^: calculated: 245.0937; observed: 245.0935.


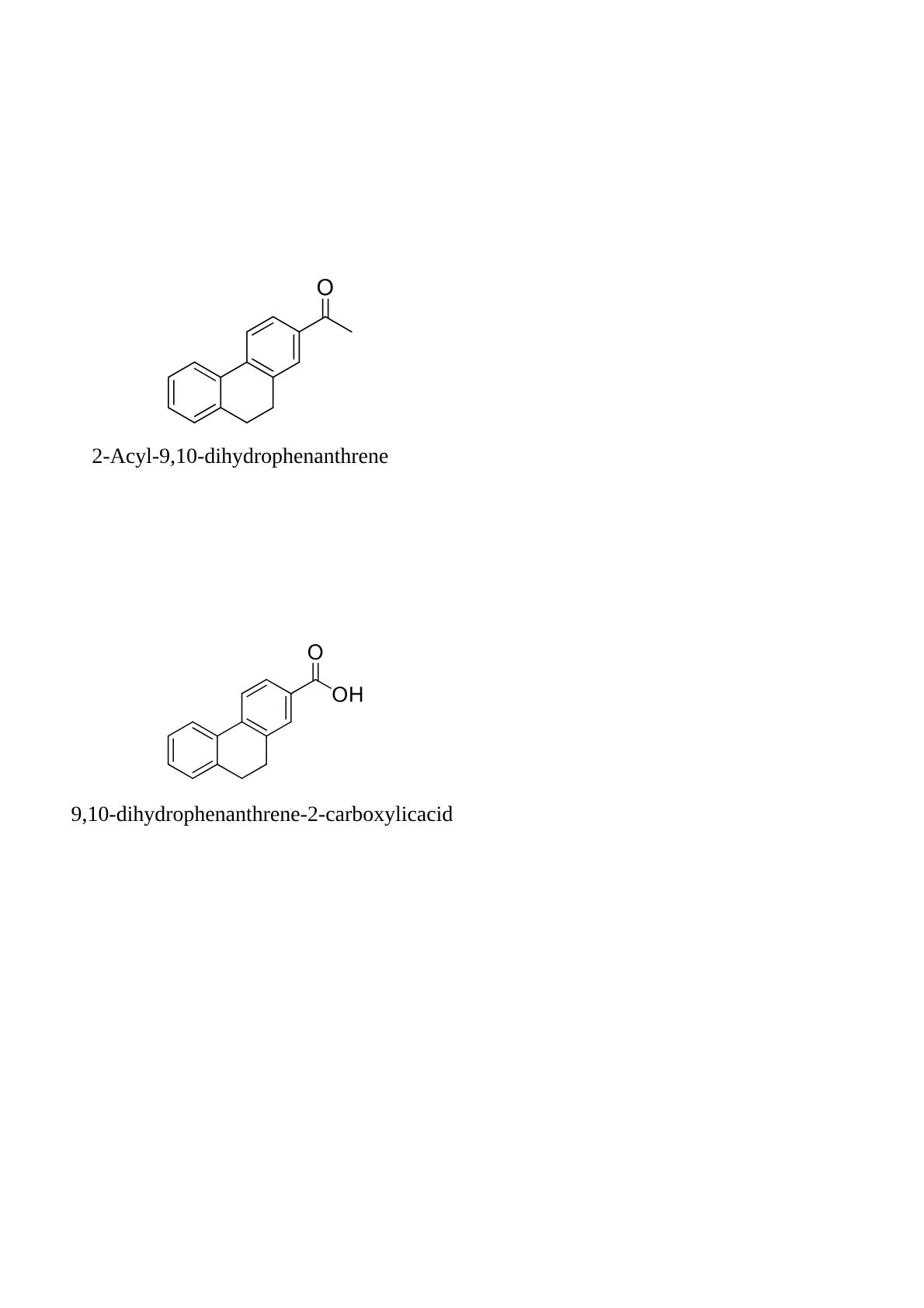


**A**

**B**

^1^H NMR (a) and ^13^C NMR (b) spectra of the chemically synthesized compound 2-acyl-9,10-dihydrophenanthrene

**Synthesis of 9,10-dihydrophenanthrene-2-carboxylic acid.** Compound 2-acyl-9,10-dihydrophenanthrene (78 mg, 0.35 mmol, 1.00 eq) was dissolved in 5 mL 1,4-dioxane and warmed to 70 °C in an oil bath. The aqueous solutions of sodium hypochlorite (1.3 m, 5.2 mL, 7.02 mmol, 20.00 eq) and sodium hydroxide (1 m, 1.1 mL, 1.03 mmol, 3.00 eq) were added dropwise. After stirring at 70 °C for 2 hours the reaction mixture was cooled to room temperature and the 1,4-dioxane removed *in vacuo*. The aqueous residue was cooled in an ice bath and carefully acidified (pH = 1) by dropwise addition of 1 M hydrochlorid acid. The precipitate was filtered off, washed three times with water and subsequently dissolved in methanol. Removing the solvent *in vacuo* yielded 9,10-dihydrophenanthrene-2-carboxylic acid (66 mg, 0.30 mmol, 86%) as a pale yellow solid. M.p.: 225 °C (decomposition). ^1^H NMR (600 MHz, CD_3_OD): δ = 7.95 (dd, ^3^*J*_H,H_ = 8.2 Hz, ^4^*J*_H,H_ = 1.8 Hz, 1H, C_ar_H), 7.91–7.83 (m, 3H, C_ar_H), 7.34–7.27 (m, 3H, C_ar_H), 2.93–2.90 (m, 2H, CH_2_), 2.89–2.87 (m, 2H, CH_2_) ppm. ^13^C NMR (151 MHz, CD_3_OD): δ = 170.0 (q, COOH), 140.3 (q, C_ar_COOH), 139.3 (q, C_ar_), 138.7 (q, C_ar_), 134.8 (q, C_ar_), 133.6 (q, C_ar_), 130.4 (t, C_ar_H), 129.6 (t, C_ar_H), 129.6 (t, C_ar_H), 129.3 (t, C_ar_H), 128.3 (t, C_ar_H), 125.4 (t, C_ar_H), 124.7 (t, C_ar_H), 30.0 (s, CH_2_), 29.9 (s, CH_2_) ppm. HRMS (ESI): [C_15_H_12_O_2_+H]^+^: calculated: 225.0910; observed: 225.0912. [C_15_H_12_O_2_+Na]^+^: calculated: 247.0730; observed: 247.0731.


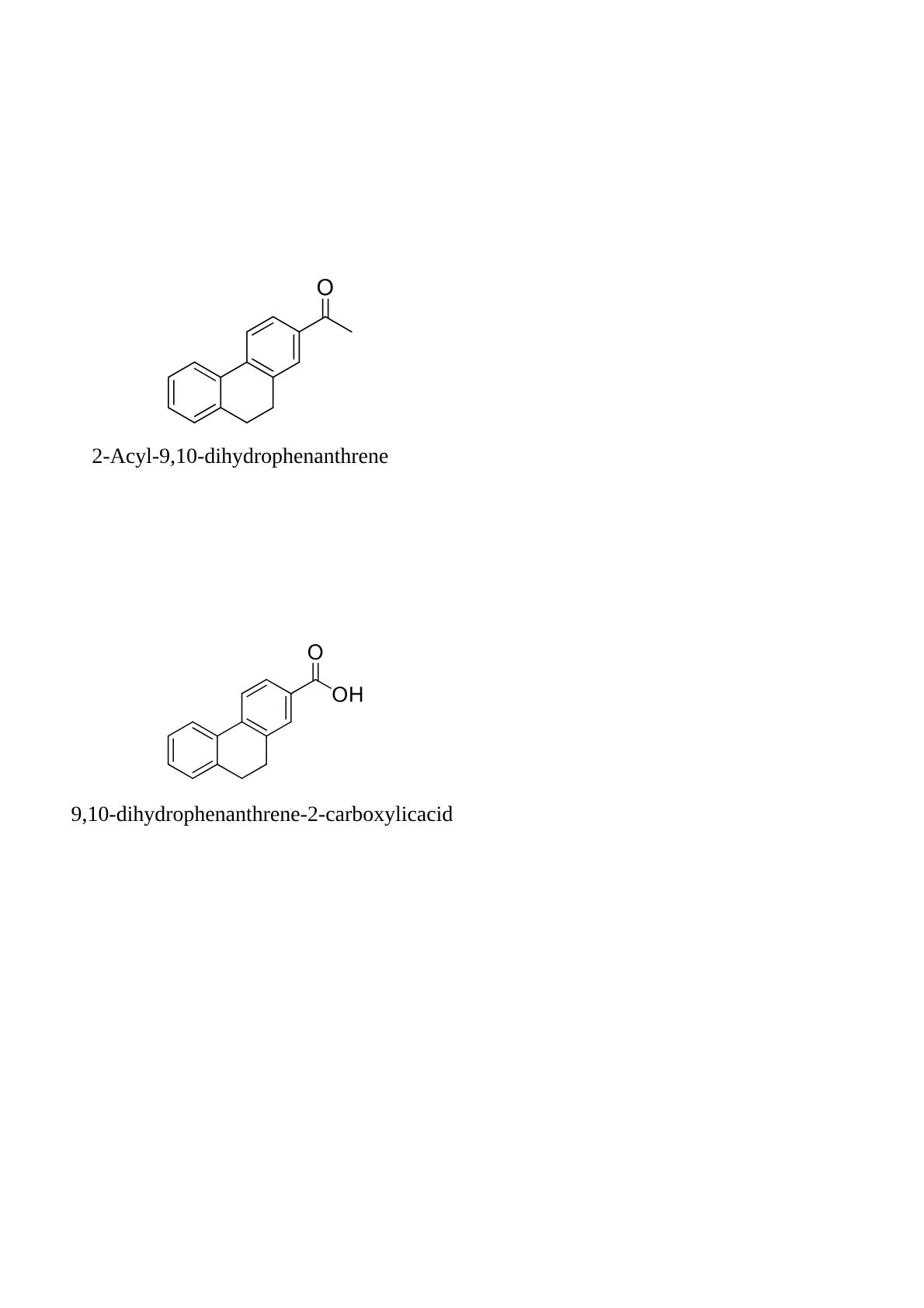


**A**

**B**

^1^H NMR (a) and ^13^C NMR (b) spectra of the chemically synthesized compound 9,10-dihydrophenanthrene-2-carboxylic acid.

**Conversion of 9,10-dihydrophenanthrene-2-carboxylic acid to 9,10-dihydro-2-phenanthroyl-CoA (isomer 3)**

9,10-dihydro-2-phenanthroyl-CoA was obtained using the same method for the conversion of 2-phenanthroic acid to 2-phenanthroyl-CoA mentioned in the main manuscript, only the 2-phenanthroic acid was replaced by 9,10-dihydrophenanthrene-2-carboxylic acid.

**REFERENCES**

1. Salentin S, Schreiber S, Haupt VJ, Adasme MF, Schroeder M. 2015. PLIP: Fully Automated Protein–Ligand Interaction Profiler. Nucleic Acids Res 43:W443-W447.

2. Adasme MF, Linnemann KL, Bolz SN, Kaiser F, Salentin S, Haupt V J, Schroeder M. 2021. PLIP 2021: Expanding the Scope of the Protein–Ligand Interaction Profiler to DNA and RNA. Nucleic Acids Res 49:W530-W534.
